# Dupilumab attenuates the expression of TSLP and IL-8 induced by dsRNA and IL-4/IL-13 co-stimulation in human small airway epithelial cells

**DOI:** 10.1371/journal.pone.0341562

**Published:** 2026-01-23

**Authors:** Aditya Sri Listyoko, Ryota Okazaki, Tomoya Harada, Genki Inui, Hiroki Kohno, Miyu Nishigami, Miki Takata, Masato Morita, Akira Yamasaki

**Affiliations:** 1 Department of Multidisciplinary Internal Medicine, Division of Respiratory Medicine and Rheumatology, Faculty of Medicine, Tottori University, Yonago, Japan; 2 Department of Pulmonology and Respiratory Medicine, Faculty of Medicine, Brawijaya University-Dr. Saiful Anwar General Hospital, Malang, Indonesia; Indian Institute of Chemical Technology, INDIA

## Abstract

**Background:**

The interaction between viral components and type 1 or type 2 cytokines during asthma exacerbations in the airway epithelium may contribute to worsening inflammation. However, these interactions in the small airway epithelium—particularly those involving alarmins (TSLP, IL-25, and IL-33) and IL-8—remain unclear. Dupilumab, a biologic agent used in severe asthma, blocks IL-4 receptor alpha (IL-4Rα) and may offer therapeutic benefits in virus-induced asthma exacerbations. In this study, we evaluate the effects of double-stranded RNA (dsRNA), in combination with various cytokines and dupilumab, on the Human Small Airway Epithelial Cells (HSAECs) line.

**Methods:**

Primary HSAECs were preincubated with dsRNA to induce the gene and protein expression of alarmins and IL-8. To evaluate the effects of cytokines on dsRNA-induced alarmin and IL-8 expression, various type 1 and type 2 cytokines were co-stimulated with dsRNA. Dupilumab was used as a pretreatment prior to co-stimulation with dsRNA and IL-4 or IL-13. Gene expression of TSLP, IL-25, IL-33, and IL-8 was assessed by quantitative PCR, and protein expression was evaluated by Western Blotting.

**Results:**

dsRNA significantly increased the expression of TSLP and IL-8. IL-4 and IL-13 further enhanced dsRNA-induced TSLP and IL-8 gene and protein expression. In contrast, TNF-α reduced dsRNA-induced TSLP expression but enhanced IL-8 gene and protein expression. Dupilumab attenuated the expression of TSLP and IL-8 induced by co-stimulation with dsRNA and IL-4 or IL-13 in HSAECs.

**Conclusion:**

In the microenvironment of small airway epithelial cells, particularly during viral infections, the presence of IL-4 or IL-13 may enhance the expression of TSLP and IL-8. Dupilumab attenuates this expression, potentially offering additional benefits in the treatment of asthma, especially during virus-induced asthma exacerbations.

## Introduction

Asthma is a prevalent respiratory condition that affects millions globally. While many individuals experience mild symptoms, the condition can become life-threatening during severe exacerbations. An asthma exacerbation is an episode of progressively worsening clinical symptoms accompanied by decreased lung function [1]. Asthma exacerbations are associated with hospitalization, readmission, reduced quality of life, increased healthcare costs, higher mortality, and an increased risk of future exacerbations [2–6]. Viral infections are the most common trigger of asthma exacerbations in both adults and children, with 52%–65% of patients experiencing exacerbations showing evidence of an underlying viral infection [7].

The airway epithelium serves as the first line of defense in the respiratory tract against pathogens and harmful environmental agents [8]. Stimulation of the airway epithelium by pathogens, such as viral components, can trigger the production of alarmins and various cytokines. A previous study reported a significant increase in thymic stromal lymphopoietin (TSLP) and interleukin-8 (IL-8) levels in bronchial epithelial cells following stimulation with double-stranded RNA (dsRNA), which acts as a viral mimic [9]. In virus-induced asthma exacerbations, interactions between Th1 and Th2 cytokines within the epithelial microenvironment may intensify inflammation and further elevate alarmin and epithelial-derived cytokine levels. However, the precise role of Th1 or Th2 cytokine interactions in the induction of alarmins or epithelial cytokines by dsRNA remains poorly understood, particularly in small airway epithelial cells.

Dupilumab is a biologic agent used in the management of severe asthma that remains uncontrolled despite treatment with medium- to high-dose inhaled corticosteroids (ICS) [1]. Dupilumab is a human monoclonal antibody that targets the interleukin-4 receptor alpha (IL-4Rα) subunit, a shared component of both IL-4 and IL-13 receptors. By blocking the binding of IL-4 and IL-13 to IL-4Rα, dupilumab inhibits downstream signaling pathways that mediate type 2 inflammation [10]. Dupilumab is currently recommended as an add-on therapy for severe asthma, with type 2 inflammation and without type 2 inflammation those taking maintenance corticosteroid [11]. However, there are no established recommendations for its use as an add-on treatment specifically for asthma exacerbations, particularly those triggered by viral infections. In this study, we hypothesized that dsRNA may enhance the expression of alarmins and IL-8 in human small airway epithelial cells. We further hypothesized that interactions with Th1 or Th2 cytokines may amplify dsRNA-induced production of alarmins and IL-8. Additionally, we investigated the effect of dupilumab on dsRNA-induced alarmin and IL-8 expression.

## Materials and methods

### Reagents

Primary human small airway epithelial cells (HSAECs) were purchased from ATCC (Manassas, USA). Recombinant human cytokines were applied to HSAECs at the following concentrations for stimulation: IL-4 (10 ng/mL), IL-5 (10 ng/mL), and IL-13 (10 ng/mL) (FUJIFILM Wako, Japan); TNF-α (10 ng/mL), CXCL-8 (10 ng/mL) (R&D Systems, Minneapolis, MN, USA), and leukotriene B4 (LTB4; 10 ⁻ ⁷ M) (Cayman Chemical, Michigan, USA). Polyinosinic-polycytidylic acid [poly(I:C)], a synthetic dsRNA analog (Tocris Bioscience, UK), was used at 10 μg/mL for quantitative polymerase chain reaction (qPCR) analysis and 25 μg/mL for Western blotting. Dupilumab (Selleck, Japan) was pre-administered at concentrations ranging from 10 to 1000 ng/mL.

### Culture and stimulation of human small airway epithelial cells (HSAECs)

Primary HSAECs were cultured in bronchial epithelial growth medium supplemented with 1.25 mL HLL supplement [final concentrations: human serum albumin (HSA) 500 mg/mL, linoleic acid 0.6 mM, lecithin 0.6 mg/mL], 15 mL L-glutamine (final concentration: 6 mM), 2 mL Extract P (final concentration: 0.4%), and 5 mL airway epithelial cell supplement [final concentrations: epinephrine 1 µM, transferrin 5 mg/mL, triiodothyronine (T3) 10 nM, hydrocortisone 5 mg/mL, recombinant human epidermal growth factor (rhEGF) 5 ng/mL, and recombinant human insulin 5 mg/mL]. The medium was further supplemented with penicillin (100 µg/mL) and streptomycin (100 µg/mL). Cells were seeded at a density of approximately 5,000 cells/cm² (∼10⁵ cells per 60 mm culture dish) and maintained at 37°C in a humidified atmosphere containing 5% CO₂ until reaching approximately 80% confluence. The culture medium was refreshed prior to stimulation. Cells were stimulated with IL-4, IL-5, IL-13, CXCL-8, TNF-α, or LTB4 for 24 hours, followed by stimulation with dsRNA for either 8 hours for qPCR analysis or 24 hours for Western blotting. Dupilumab was administered as a pre-treatment at concentrations ranging from 10 to 1000 ng/mL for 8 hours prior to cytokine stimulation.

### Real-time quantitative PCR

Cells were lysed directly using RLT buffer supplemented with β-mercaptoethanol. Total RNA was extracted using the RNeasy Plus Mini Kit (Qiagen, Hilden, Germany) according to the manufacturer’s protocol. Total RNA concentrations were measured using a NanoDrop spectrophotometer. Complementary DNA (cDNA) was synthesized from equal amounts of RNA using the SuperScript™ IV VILO™ Master Mix (Invitrogen, Thermo Fisher Scientific, Waltham, USA), following the recommended reverse transcription protocol. Quantitative real-time PCR was conducted using specific primers for TSLP, IL-25, IL-33, and IL-8 (Integrated DNA Technologies, Coralville, USA). Reactions were performed using SYBR™ Green PCR Master Mix (Applied Biosystems, Thermo Fisher Scientific, Waltham, USA) on a ViiA™ 7 Real-Time PCR System. Gene expression levels were quantified using the 2 ⁻ ΔΔCt method and normalized to GAPDH as the reference housekeeping gene. The non-stimulated control group was used as the calibrator for relative gene expression analysis. No universal cut-off value exists for 2 ⁻ ΔΔCt; therefore, results are reported as fold changes relative to the non-stimulated control group. Statistical significance was determined based on biological replicates and appropriate statistical analyses.

### Cell lysate preparation, protein quantification, and western blotting

Cells were washed with ice-cold phosphate-buffered saline (PBS) and lysed using radioimmunoprecipitation assay (RIPA) buffer composed of 50 mM Tris-HCl (pH 7.4), 1% NP-40, 0.25% sodium deoxycholate, 150 mM NaCl, 1 mM EDTA, 1 mM sodium orthovanadate (Na₃VO₄), 1 mM sodium fluoride (NaF), and a protease inhibitor cocktail. Protein concentrations were quantified using a protein assay, and equal amounts of total protein were separated by Tris-glycine SDS-polyacrylamide gel electrophoresis (SDS-PAGE). Proteins were then transferred onto polyvinylidene difluoride (PVDF) membranes (Amersham Hybond-P, GE Healthcare Life Sciences, Buckinghamshire, UK). Membranes were blocked with 3% bovine serum albumin (BSA) in Tris-buffered saline containing 0.1% Tween-20 (TBS-T; 20 mM Tris, 150 mM NaCl, pH 7.6) for 1 hour at room temperature. Following blocking, membranes were incubated overnight at 4°C in 5% BSA in TBS-T containing the following primary antibodies: anti-TSLP (1:1000, GeneTex, Irvine, USA), anti-IL-33 (1:1000, GeneTex, Irvine, USA), anti-IL-17E (1:1000, GeneTex, Irvine, USA), and anti-IL-8 (1:500, Santa Cruz Biotechnology, Dallas, USA). After washing, membranes were incubated for 1 hour at room temperature with horseradish peroxidase (HRP)-conjugated secondary antibodies diluted 1:2000 in 2% BSA-TBS-T (anti-rabbit IgG or anti-mouse IgG as appropriate). Protein bands were visualized using enhanced chemiluminescence (ECL) reagents (GE Healthcare Life Sciences) on an ImageQuant LAS 4000 mini. Densitometric analysis was performed using TotalLab Quant software version 7.0 (TotalLab Ltd., Newcastle, UK). Protein expression levels were quantified by densitometric analysis and normalized to β-actin as the reference loading control. The non-stimulated control group was used as the calibrator for relative protein expression analysis. As no universal cut-off value exists for densitometric quantification, results are reported as fold changes relative to the non-stimulated control group. Statistical significance was determined based on biological replicates and appropriate statistical analyses.

### Statistical analysis

Data are presented as mean ± standard deviation (SD). Statistical comparisons were performed using one-way analysis of variance (ANOVA), followed by appropriate post hoc multiple comparison tests. A p-value < 0.05 was considered statistically significant. All analyses were performed using GraphPad Prism, version 10.4.1

## Results

### dsRNA induces the gene and protein expression of TSLP and IL-8

**Fig 1:**
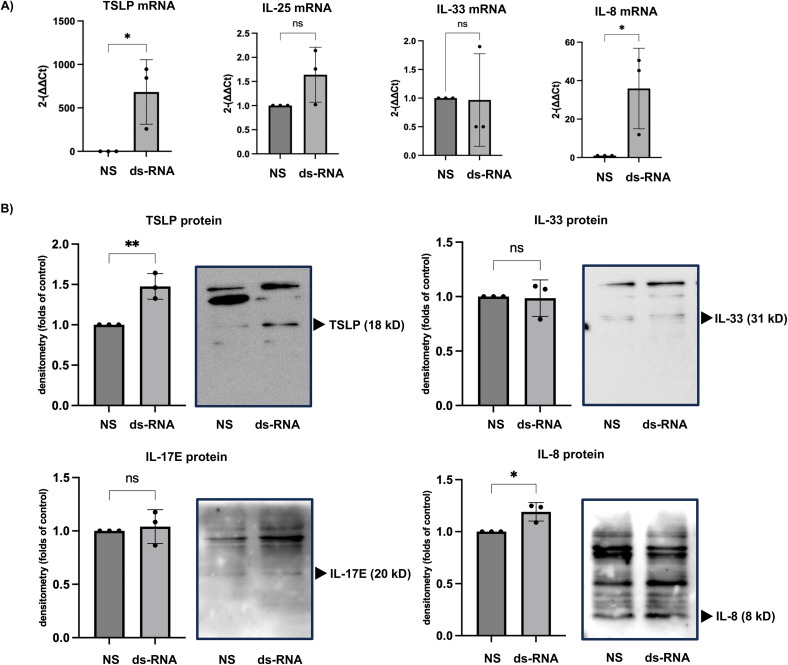
dsRNA-Induced TSLP and IL-8 Gene and Protein Expression in the HSAECs. (A) Relative changes in gene expression show that dsRNA stimulation markedly increased TSLP and IL-8 mRNA levels compared with the non-stimulated control group. (B) Densitometric analysis demonstrates that dsRNA stimulation similarly increased TSLP and IL-8 protein expression compared with the non-stimulated control group. Results are presented as fold changes relative to the non-stimulated control group and expressed as the mean ± standard deviation (SD) from at least three independent experiments. Statistical significance (*: p < 0.05, **: p < 0.005) was determined compared to the non-stimulated group.

Stimulation with dsRNA significantly increased the expression of TSLP and IL-8 genes. Notably, TSLP gene expression increased by an average of 682.8-fold compared to the non-stimulated group, while IL-8 expression increased by an average of 35.91-fold. In contrast, dsRNA stimulation did not induce the expression of IL-25 or IL-33 genes. Consistent with the gene expression findings, dsRNA stimulation also increased TSLP and IL-8 protein levels compared with the non-stimulated control group. Similarly, IL-17E and IL-33 protein levels remained unchanged following dsRNA stimulation, aligning with the gene expression results (Fig 1).

### Co-stimulation with IL-4 and IL-13 enhances dsRNA-induced TSLP and IL-8 gene and protein expression

**Fig 2:**
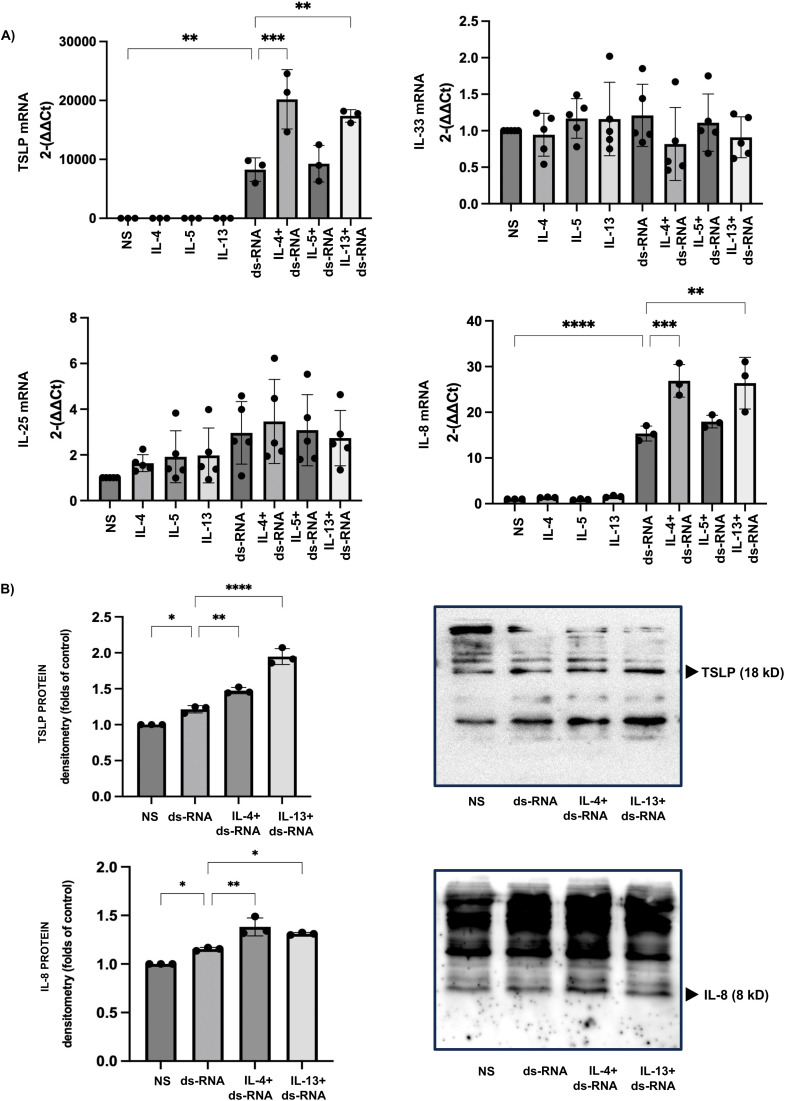
IL-4 and IL-13 Enhance dsRNA-Induced TSLP and IL-8 Gene and Protein Expression in the HSAECs. (A) Relative changes in gene expression show that dsRNA stimulation markedly increased TSLP and IL-8 mRNA levels compared with the non-stimulated control group. Co-stimulation with IL-4 or IL-13 further enhanced dsRNA-induced TSLP and IL-8 gene expression compared with the dsRNA-only group. (B) Densitometric analysis demonstrates that co-stimulation with IL-4 or IL-13 similarly increased TSLP and IL-8 protein expression compared with the dsRNA group. Results are presented as fold changes relative to the non-stimulated control group and expressed as the mean ± standard deviation (SD) from at least three independent experiments. Results are presented as fold changes relative to the non-stimulated control group and expressed as the mean ± standard deviation (SD) from at least three independent experiments. Statistical significance (*: p < 0.05, **: p < 0.005, ***: p < 0.001, ****: p < 0.0001) was evaluated compared to the non-stimulated or dsRNA-only group.

Treatment with Th2 cytokines alone (IL-4, IL-5, or IL-13) did not induce the expression of TSLP, IL-25, IL-33, or IL-8 in HSAECs. However, co-stimulation with IL-4 or IL-13 significantly enhanced dsRNA-induced TSLP expression (relative TSLP expression: dsRNA group, 8269 ± 2006; dsRNA + IL-4 group, 20,198 ± 5034; dsRNA + IL-13 group, 17,385 ± 1063) and IL-8 expression (relative IL-8 expression: dsRNA group, 15.36 ± 1.63; dsRNA + IL-4 group, 26.87 ± 3.57; dsRNA + IL-13 group, 26.37 ± 5.66). In contrast, IL-5 did not augment dsRNA-induced TSLP or IL-8 expression. Consistent with the gene expression results, co-stimulation with IL-4 or IL-13 also increased TSLP and IL-8 protein levels compared with dsRNA stimulation alone (Fig 2).

### Co-stimulation with TNF-α suppresses dsRNA-induced TSLP expression while enhancing IL-8 gene and protein expression

**Fig 3:**
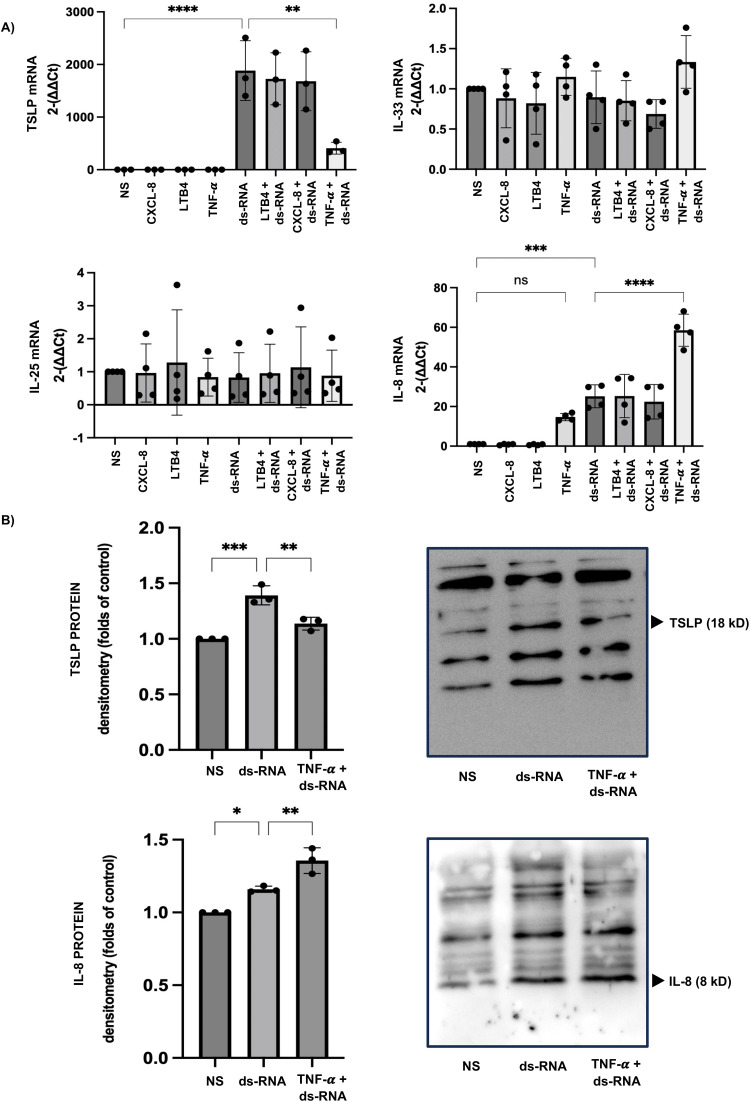
TNF-α Reduces dsRNA-Induced TSLP Expression but Enhances IL-8 Gene and Protein Expression in the HSAECs. (A) Relative changes in gene expression show that dsRNA stimulation markedly increased TSLP and IL-8 mRNA levels compared with the non-stimulated control group. Co-stimulation with IL-4 or IL-13 further enhanced dsRNA-induced TSLP and IL-8 gene expression compared with the dsRNA-only group. (B) Densitometric analysis demonstrates that co-stimulation with IL-4 or IL-13 similarly increased TSLP and IL-8 protein expression compared with the dsRNA group. Results are presented as fold changes relative to the non-stimulated control group and expressed as the mean ± standard deviation (SD) from at least three independent experiments. Statistical significance (*: p < 0.05, **: p < 0.005, ***: p < 0.001, ****: p < 0.0001) was evaluated compared to the non-stimulated or dsRNA-only group.

Stimulation with CXCL-8 or LTB4 alone did not induce the expression of TSLP, IL-8, IL-25, or IL-33. Similarly, TNF-α alone did not induce TSLP, IL-25, or IL-33, although it did increase IL-8 gene expression (relative IL-8 expression: TNF-α group, 14.69 ± 1.84, compared with the non-stimulated control group). Co-stimulation with CXCL-8 or LTB4 and dsRNA did not enhance TSLP or IL-8 expression. In contrast, co-stimulation with TNF-α and dsRNA suppressed dsRNA-induced TSLP gene expression (relative TSLP expression: dsRNA group, 1884 ± 567.20; dsRNA + TNF-α group, 408.9 ± 112.20) while further increasing IL-8 gene expression (relative IL-8 expression: dsRNA group, 25.16 ± 5.80; dsRNA + TNF-α group, 58.53 ± 8.12). These gene expression findings were consistent with protein-level results, as TNF-α co-stimulation reduced TSLP protein levels and enhanced IL-8 protein expression compared with dsRNA stimulation alone (Fig 3).

### Dupilumab attenuates TSLP and IL-8 expression induced by co-stimulation with dsRNA and IL-4

**Fig 4:**
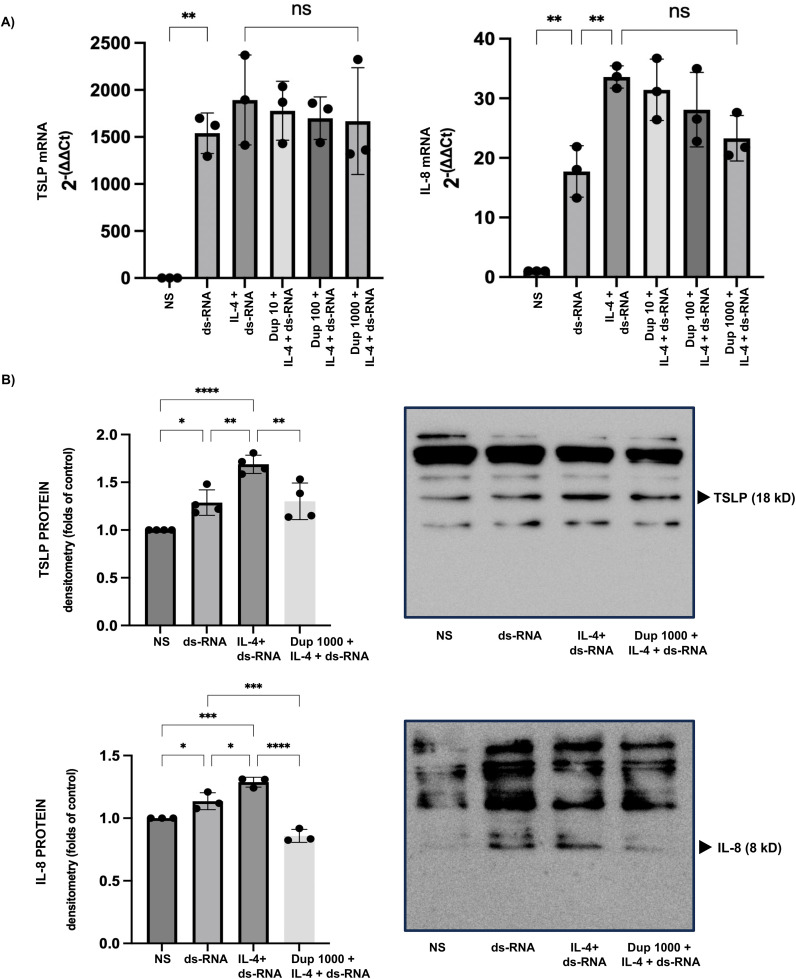
Dupilumab attenuates TSLP and IL-8 expression induced by co-stimulation with dsRNA and IL-4 in the HSAECs. (A) Relative changes in gene expression show that dsRNA stimulation markedly increased TSLP and IL-8 mRNA levels compared with the non-stimulated control group. Co-stimulation with IL-4 further enhanced dsRNA-induced TSLP and IL-8 gene expression. Pretreatment with dupilumab (10–1000 ng/mL) showed a trend toward decreasing the relative expression of both TSLP and IL-8. (B) Densitometric analysis demonstrates that co-stimulation with IL-4 similarly increased TSLP and IL-8 protein levels, and this effect was attenuated by 1000 ng/mL dupilumab. Results are presented as fold changes relative to the non-stimulated control group and expressed as the mean ± standard deviation (SD) from at least three independent experiments. Statistical significance (*: p < 0.05, **: p < 0.005, ***: p < 0.001, ****: p < 0.0001) was evaluated compared to the non-stimulated or dsRNA-only group.

Dupilumab pretreatment (10–1000 ng/mL) showed a trend toward reducing TSLP gene expression induced by co-stimulation with IL-4 and dsRNA (relative TSLP expression: IL-4 + dsRNA group, 1.893 ± 478.1; dupilumab 10, 100, and 1000 ng/mL groups, 1.779 ± 313.2, 1.700 ± 225.8, and 1.669 ± 568.7, respectively). A similar trend was observed for IL-8 gene expression (relative IL-8 expression: IL-4 + dsRNA group, 33.58 ± 1.86; dupilumab 10, 100, and 1000 ng/mL groups, 31.43 ± 5.15, 28.09 ± 6.24, and 23.29 ± 3.82, respectively). The greatest reduction in both TSLP and IL-8 gene expression was observed with the highest dupilumab concentration (1000 ng/mL), although these reductions did not reach statistical significance. Furthermore, protein expression analysis demonstrated that pretreatment with 1000 ng/mL dupilumab significantly decreased TSLP and IL-8 protein levels compared with the IL-4 + dsRNA group (Fig 4).

### Dupilumab attenuates TSLP and IL-8 expression induced by co-stimulation with dsRNA and IL-13

**Fig 5:**
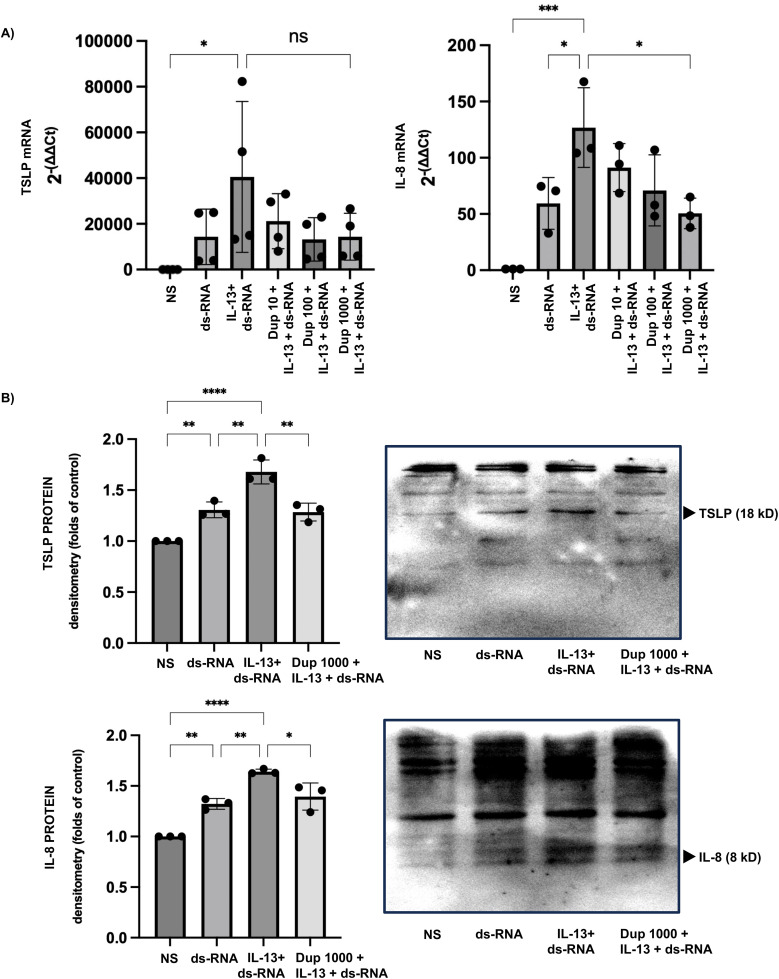
Dupilumab attenuates TSLP and IL-8 expression induced by co-stimulation with dsRNA and IL-13 in the HSAECs. (A) Relative changes in gene expression show that dsRNA stimulation markedly increased TSLP and IL-8 mRNA levels compared with the non-stimulated control group. Co-stimulation with IL-13 further enhanced dsRNA-induced TSLP and IL-8 gene expression. Pretreatment with dupilumab (10–1000 ng/mL) showed a trend toward decreasing the relative expression of both TSLP and IL-8. (B) Densitometric analysis demonstrates that co-stimulation with IL-13 similarly increased TSLP and IL-8 protein levels, and this effect was attenuated by 1000 ng/mL dupilumab. Results are presented as fold changes relative to the non-stimulated control group and expressed as the mean ± standard deviation (SD) from at least three independent experiments. Statistical significance (*: p < 0.05, **: p < 0.005, ****: p < 0.0001) was evaluated compared to the non-stimulated or dsRNA-only group.

Dupilumab pretreatment (10–1000 ng/mL) demonstrated a trend toward reducing TSLP gene expression induced by co-stimulation with IL-13 and dsRNA (relative TSLP expression: IL-13 + dsRNA group, 40,519 ± 33,001; dupilumab 10, 100, and 1000 ng/mL groups, 21,205 ± 12,043; 13,222 ± 9,512; and 14,387 ± 10,227, respectively). A similar trend was observed for IL-8 gene expression, with 1000 ng/mL dupilumab significantly suppressing IL-8 levels (relative IL-8 expression: IL-13 + dsRNA group, 126.8 ± 35.43; dupilumab 10, 100, and 1000 ng/mL groups, 91.29 ± 21.38; 70.98 ± 31.54; and 50.52 ± 13.59, respectively). Furthermore, protein expression analysis revealed that pretreatment with 1000 ng/mL dupilumab significantly reduced both TSLP and IL-8 protein levels compared with the IL-13 + dsRNA group. (Fig 5).

## Discussion

In the present study, we observed that Poly I:C, a synthetic analog of dsRNA, selectively modulated the expression of epithelial alarmins in HSAECs. Specifically, Poly I:C significantly upregulated TSLP expression, while it had no apparent effect on IL-25 or IL-33. In addition, Poly I:C stimulation enhanced IL-8 expression. These findings suggest that Poly I:C induces a selective release of proinflammatory alarmins and cytokines in small airway epithelial cells. Previous studies have reported that Poly I:C upregulates TSLP and IL-8 expression in keratinocytes [12], TSLP in bronchial epithelial cells [13], TSLP in human primary laryngeal arytenoid fibroblast [14], IL-8 in human nasal fibroblasts [15], and IL-33 in human lung-derived microvascular endothelial cells [16], bronchial smooth muscle cells [17], and oligodendrocyte precursor cells [18]. Our study provides additional insight into how external stimuli, such as viral particles, can trigger not only alarmin release and innate immune responses in the large airways (e.g., the bronchi), but also induce the expression of alarmins—particularly TSLP—and pro-inflammatory cytokines like IL-8 in the small airway epithelium. This finding may be important in the context of asthma, as it supports the idea that the small airways also play a role in the immune response to external stimuli and may contribute to the pathogenesis of asthma [19,20].

Poly I:C is widely used in research to mimic viral infections and activate innate immune responses. It primarily functions by stimulating Toll-like receptor 3 (TLR3), leading to the induction of epithelial-derived alarmins, type I interferons, and pro-inflammatory cytokines [12,21,22]. However, the use of Poly I:C as a model for virus-induced asthma exacerbation has certain limitations. While Poly I:C is particularly effective for investigating innate immune responses—especially in epithelial cells exposed to external stimuli—it does not fully replicate the complex pathophysiology of infections caused by clinically relevant respiratory viruses that frequently trigger asthma exacerbations, most of which are single-stranded RNA viruses, such as rhinovirus, respiratory syncytial virus (RSV), and influenza virus [7]. Nevertheless, Poly I:C remains a valuable experimental stimulus in in vitro models of virus-induced asthma exacerbation, as it functions as a TLR3 ligand. Moreover, viruses such as rhinovirus, RSV, and influenza can trigger inflammatory cascades that may be partially regulated by TLR3 signaling [12,21,23–25].

In our study, treatment with 10 μg/mL and 25 μg/mL of Poly I:C did not induce IL-25 or IL-33 gene or protein expression in human small airway epithelial cells. These findings are consistent with the study by Choi et al., which demonstrated that Poly I:C stimulation did not induce IL-25 or IL-33 production in BEAS-2B epithelial cells [26]. In contrast, a previous study using human nasal epithelial cells reported that Poly I:C at concentrations between 10 and 75 μg/mL significantly upregulated both IL-25 and IL-17F at the gene and protein levels [27]. Interestingly, in a mouse model of virus-induced asthma exacerbation, administration of Poly I:C induced IL-33 gene expression, but not IL-25, in wild-type mice. Notably, this increase in IL-33 expression was absent in IL-1β–deficient mice, suggesting that IL-1β may play a critical role in dsRNA-induced IL-33 upregulation in this context [28]. These findings suggest that alarmin responses may vary depending on the cell type and the nature of the stimulus used to induce their release. In airway epithelial cells, stimulation with Poly I:C does not significantly promote the release of IL-25 or IL-33. In contrast, the release of these alarmins is typically more pronounced in allergen-induced asthma models, such as those involving stimulation with house dust mite or Alternaria alternata extracts [29,30].

Our study demonstrated that among the Th2 cytokines, the presence of IL-4 or IL-13 significantly enhanced the dsRNA-induced release of TSLP and IL-8 in HSAECs. A similar finding was reported by Kinoshita et al., who observed that IL-4 and IL-13, in combination with TNF-α, exerted a synergistic effect in upregulating TSLP and IL-8 expression in keratinocyte cells [12]. IL-4 has also been shown to enhance TSLP expression in a mouse model of keratitis induced by *Aspergillus fumigatus* [31]. Combined stimulation with IL-4 and dsRNA has also been shown to increase TSLP production in normal human bronchial epithelial cells [32]. IL-13 has also been observed to induce TSLP expression in mouse nasal tissue specimens [33], suggesting that both IL-4 and IL-13 may interact in a feedback loop to promote TSLP production. IL-4 and IL-13 are closely related cytokines derived from Th2 cells that play pivotal roles in allergic inflammation by promoting the recruitment and activation of IgE-producing B cells and amplifying IgE-mediated immune responses [34,35]. During rhinovirus infection, type 2 cytokines—including IL-4 and IL-13—were significantly elevated in both nasal and bronchial samples of asthma patients [36,37]. An in vitro study also demonstrated increased expression of IL-33 and type 2 cytokines—IL-4, IL-5, and IL-13—in RV16-infected bronchial epithelial cells. This effect was abolished by IL-33 blockade, suggesting that IL-33 promotes the type 2 immune response during rhinovirus infection [37]. As discussed previously, our study did not observe an increase in IL-33 following Poly I:C stimulation, which may reflect differences in epithelial alarmin responses between Poly I:C and whole virus as stimuli. Taken together, these findings suggest that during virus-induced asthma exacerbations, viral infection induces epithelial alarmins—particularly IL-33—which act upstream to trigger Th2 cytokine release. The presence of Th2 cytokines, especially IL-4 and IL-13, may in turn amplify a feedback loop that enhances TSLP and IL-8 release from epithelial cells, potentially contributing to worsening disease outcomes. Furthermore, our study found that IL-5 did not enhance TSLP or IL-8 release in HSAECs. This finding may reflect the specific biological role of IL-5, which primarily functions as a key mediator of eosinophil differentiation, growth, activation, survival, and recruitment to the airways, rather than directly influencing the biological functions of epithelial cells [38].

Our study also investigated mediators beyond Th2 cytokines to assess potential synergistic effects with dsRNA, including chemokines and cytokines associated with Th1 responses, such as CXCL8, the lipid mediator leukotriene B4, and the Th1 cytokine TNF-α. We hypothesized that Th1 cytokines also modulate alarmin and cytokine release induced by dsRNA in epithelial cells. A previous study by Okuma et al. reported that pretreatment with IFN-γ enhanced Poly I:C-induced IL-6 production in NCI-H292 bronchial epithelial cells [39]. We observed that TNF-α suppressed dsRNA-induced TSLP release while enhancing IL-8 production. The attenuation of TSLP expression by TNF-α may be attributed to TNF-α–induced damage in normal small airway epithelial cells, leading to a reduced production of epithelial-derived alarmins such as TSLP. Potential mechanisms underlying this epithelial damage include necrosis, apoptosis, and disruption of cell–cell contacts. Moreover, TNF-α may contribute to mitochondrial dysfunction in bronchial epithelial cells, as suggested by a reduction in glucose oxidation rates following TNF-α exposure [40]. Moreover, TNF-α alone, even in the absence of dsRNA, showed a trend toward inducing IL-8 expression. TNF-α is a proinflammatory cytokine produced by various cell types—including monocytes, fibroblasts, epithelial cells, endothelial cells, and smooth muscle cells—following stimulation. It exerts significant pathophysiological effects on both epithelial and endothelial tissues [41]. Pretreatment of epithelial cells with TNF-α has been shown to enhance virus-induced chemokine responses and induce the activation of transcription factors such as IRF1, IRF7, and the p50 subunit of NF-κB. These findings suggest that in virus-infected epithelial cells, the presence of TNF-α may amplify the production of cytokines and chemokines [42]. Our study showed similar findings to previous reports, demonstrating that TNF-α enhances IL-8 production in RSV-infected A549 epithelial cells. This suggests that TNF-α may act in an autocrine manner to stimulate or enhance the production of other cytokines, such as IL-6 and CXCL8 [43–45]. Interestingly, an animal model of allergic airway disease demonstrated that RSV infection increases the production of TNF-α [46]. Taken together, these findings suggest that TNF-α production is generally elevated during virus-induced asthma exacerbations. Within the epithelial microenvironment, TNF-α may contribute to epithelial cell damage, leading to a decreased production of epithelial-derived TSLP. Conversely, TNF-α may also activate transcription factors and function in an autocrine manner to promote or amplify the production of proinflammatory cytokines and chemokines, such as IL-8.

Our study observed that dupilumab attenuates the co-stimulatory effect of IL-4 or IL-13 with dsRNA on TSLP expression in HSAECs. Dupilumab is a monoclonal antibody that binds to the IL-4Rα, thereby blocking the signaling of IL-4 and IL-13—key cytokines that drive inflammation, particularly in type 2 asthma. Dupilumab is used as an add-on maintenance treatment for severe asthma that remains uncontrolled despite high-dose ICS therapy [10,11,35]. A human experimental model of rhinovirus infection demonstrated elevated expression of Th2 cytokines, including IL-4 and IL-13, which was associated with the severity of exacerbation. Moreover, rhinovirus infection of primary human bronchial epithelial cells induced IL-33 expression. Notably, culturing human T cells and group 2 innate lymphoid cells (ILC2s) with supernatants from rhinovirus-infected bronchial epithelial cells resulted in a robust induction of type 2 cytokine production [37]. In asthma mouse models, early RSV infection has also been shown to exacerbate airway inflammation and enhance Th2 cytokine expression, including IL-4 and IL-13 [47]. Virus-induced asthma exacerbations appear to involve not only the upregulation of Th2 cytokines but also an increased expression of receptors, particularly IL-4Rα, in both epithelial and CD4 ⁺ T helper cells. A previous study reported that transfection of Poly I:C into BEAS-2B epithelial cells increased the mRNA and protein expression of IL-4Rα and IL-2Rγ, which are key components of the IL-4 receptor complex [48]. Interestingly, in the context of RSV infection, mice infected with RSV showed an upregulation of IL-4Rα on CD4 ⁺ T helper cells [49]. Taken together, these findings suggest that dupilumab does not directly suppress TSLP production. Viral infection or stimulation with dsRNA can upregulate TSLP expression, an effect that is further amplified in the presence of IL-4 or IL-13. In addition, viral infection or dsRNA may enhance the expression of Th2 cytokines and the IL-4Rα complex. Dupilumab exerts its effect by binding to the IL-4 receptor complex, thereby blocking IL-4 and IL-13 signaling. Since both IL-4 and IL-13 contribute to a positive feedback loop that promotes TSLP expression, inhibition of their signaling by dupilumab may help mitigate inflammation during virus-induced asthma exacerbations by indirectly reducing TSLP production.

We also observed that dupilumab attenuated IL-8 production induced by dsRNA in the presence of IL-4 or IL-13 co-stimulation. However, the mechanism by which dupilumab reduces IL-8 production remains unclear. Similar to its effect on TSLP, dupilumab may not directly reduce IL-8 levels but rather modulate the IL-4- or IL-13-induced enhancement of IL-8 release in HSAECs. The effect of IL-4 or IL-13 on IL-8 production from epithelial cells is complex and varies depending on the specific cell type and the presence of additional stimuli. A previous study reported that IL-4 and IL-13 strongly inhibit IL-8 secretion in human intestinal epithelial cells [50], while stimulating IL-8 release in human bronchial epithelial cells [51]. Inoculation of bronchial epithelial cells with influenza virus and A549 airway epithelial cells with RSV increases IL-8 production [52,53], suggesting that abundant IL-8 release from epithelial cells is driven not only by cytokine stimulation but also directly induced by viral stimuli. A similar result was observed following inoculation with RV16 in pediatric primary bronchial epithelial cells, where IL-8 production increased, and this increase was further enhanced in the presence of Th2 cytokines [54]. Taken together, in the context of virus-induced asthma exacerbation, viral components such as dsRNA or whole viruses like influenza and RSV can induce IL-8 production from epithelial cells. This production is further augmented in the presence of Th2 cytokines such as IL-4 or IL-13. dupilumab, by competitively inhibiting IL-4 and IL-13 binding to IL-4Rα, attenuates Th2 cytokine-mediated enhancement of IL-8 production induced by dsRNA or viral infection in epithelial cells.

The induction of TSLP and IL-8 in epithelial cells in response to viral stimulation, and their interaction with Th2 cytokines in the microenvironment—particularly during viral infections in asthma—should be a focus of attention for clinicians, especially in severe asthma cases where the selection of appropriate biologic agents should be guided by the underlying pathophysiological condition. In clinical practice, the anti-TSLP agent tezepelumab may offer benefits as it acts upstream, regulating both type 1 and type 2 inflammatory pathways [55]. However, a Bayesian network meta-analysis comparing biologic agents in asthma found that both tezepelumab and dupilumab were associated with greater improvements in exacerbation rates and lung function compared with other biologic agents [56]. Although current guidelines recommend both tezepelumab and dupilumab for severe asthma, particularly eosinophilic asthma with elevated blood eosinophil counts or FeNO levels [1], our findings suggest that dupilumab may provide greater benefits than tezepelumab in virus-induced asthma exacerbations, where excessive IL-8 production occurs, as tezepelumab does not inhibit this pathway [57]. Another important clinical implication is that, during viral infections, viral components can initiate an inflammatory cascade in the peripheral or small airways. Our study demonstrated that dsRNA induces the release of TSLP and IL-8 in the small airways, and that dupilumab may provide effective inflammation control in the small airways epithelium during virus-induced exacerbations by reducing TSLP and IL-8 production. The ability of dupilumab to control inflammation in the small airways may be related to studies reporting improvements in small airway parameters following treatment with biologic agents in severe asthma [58].

**Fig 6:**
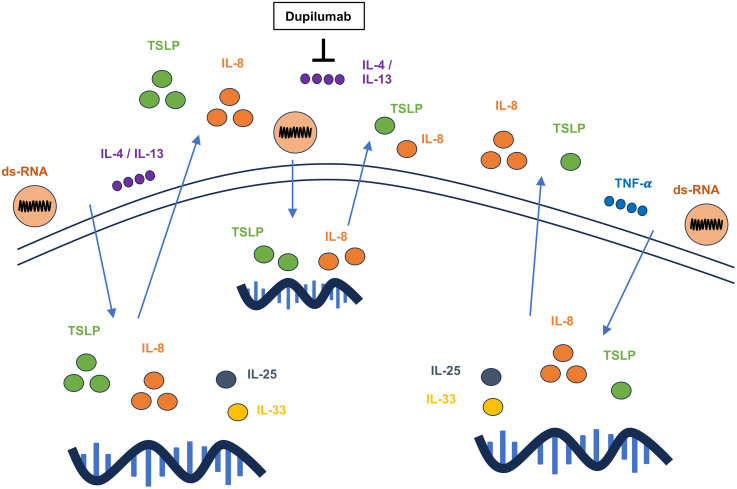
Graphical abstract illustrating the costimulatory effects of dsRNA and cytokines on alarmin and IL-8 production in HSAECs. dsRNA significantly increased the expression of TSLP and IL-8. IL-4 and IL-13 further enhanced dsRNA-induced TSLP and IL-8 gene and protein expression. In contrast, TNF-α reduced dsRNA-induced TSLP expression but enhanced IL-8 gene and protein expression. Dupilumab attenuated the expression of TSLP and IL-8 induced by co-stimulation with dsRNA and IL-4.

Our study demonstrated that dsRNA induces the release of TSLP and IL-8 in HSAECs, and this response is further augmented in the presence of IL-4 and IL-13. In contrast, TNF-α reduced dsRNA-induced TSLP production but enhanced IL-8 release. Dupilumab attenuated the expression of both TSLP and IL-8 induced by co-stimulation with dsRNA and IL-4/IL-13 in HSAECs (Fig 6), suggesting its potential therapeutic benefit in virus-induced asthma exacerbations, particularly in type 2 asthma. However, this study has several limitations. First, we did not use whole viruses typically involved in virus-induced asthma exacerbations, such as rhinovirus, RSV, or influenza, which may produce different responses in HSAECs compared to dsRNA. Second, we directly stimulated HSAECs with Th2 cytokines without evaluating whether dsRNA influences the production of these cytokines within epithelial cells. Although IL-4 and IL-13 are predominantly produced by Th2 cells, epithelial cells may also release these cytokines in response to dsRNA or viral infection. Furthermore, it remains to be determined whether dsRNA stimulation directly upregulates IL-4 or IL-13 receptor expression in HSAECs.

The data presented in our study represent an early exploratory stage, using a single epithelial cell line to examine small airway inflammation and the regulation of epithelial alarmins in response to dsRNA exposure. We propose further studies to elucidate the precise inflammatory mechanisms in small airway epithelium by silencing (siRNA/shRNA) or completely deleting (CRISPR knockout) key genes such as TSLP or IL-4Rα. These approaches are crucial for confirming their functional roles in major inflammatory pathways. For example, knocking out IL-4Rα would prevent IL-4 or IL-13 from binding and activating downstream signaling molecules such as JAK1/JAK3 and STAT6, thereby reducing TSLP production. More advanced investigations using single-cell profiling of airway tissue—particularly from patients with virus-induced asthma exacerbations—although ethically challenging, would allow precise identification of the specific cell types that express TSLP, IL-4Rα, and IL-8, as well as the signaling pathways activated within each population. Single-cell profiling, particularly scRNA-seq, can also reveal how immune cells respond to epithelial alarmins and provide detailed mapping of immune–epithelial interactions. Finally, ex vivo studies using primary small airway epithelial cells isolated from three groups—patients with severe asthma, patients with asthma receiving dupilumab, and healthy controls—are essential to determine whether the mechanisms observed in our in vitro cell line model also occur in patient-derived airway cells. If dupilumab similarly modifies dsRNA-induced TSLP and IL-8 production in these primary epithelial cells, this would substantially strengthen the clinical relevance of our proposed mechanistic pathway model. We note that dupilumab has not yet been established as an add-on treatment in the initial management of asthma exacerbations. However, applying this approach in patients with severe asthma who are receiving dupilumab and performing cellular profiling—particularly in those with a Th2-high allergic phenotype—could help clarify the role of dupilumab in real-world settings. Specifically, such profiling studies could determine whether dupilumab can attenuate the increased release of epithelial-derived mediators, such as TSLP or IL-8, in patients with established type 2 inflammation.

Exploring the pathomechanisms of virus-induced asthma exacerbations and developing precision medicine approaches targeting virus-induced cytokine responses may be of substantial importance in the future. Virus-induced asthma exacerbations are predominantly characterized by neutrophilic airway inflammation with increased IL-8 expression and are often associated with more severe clinical outcomes, including higher hospitalization rates and prolonged recovery times [59–62]. From a clinical diagnostic perspective, accurate identification of the causative virus requires specific detection methods, such as PCR testing of nasal or sputum samples [63]. Moreover, when neutrophilic inflammation predominates, accompanied by elevated IL-8 levels, these exacerbations may be less responsive to standard inhaled corticosteroid therapy. Our study highlights an important aspect of virus-induced asthma exacerbations. In patients with asthma who have a pre-existing tendency toward allergic (Th2-skewed) inflammation, the presence of Th2 cytokines—particularly IL-4 and IL-13—amplifies the release of TSLP and IL-8 from small airway epithelial cells. Under these conditions, dupilumab may attenuate this exaggerated inflammatory response and may be associated with clinical improvement during virus-induced asthma exacerbations.

## Conclusion

Within the microenvironment of small airway epithelial cells, particularly during viral infections, the presence of IL-4 or IL-13 may augment the expression of TSLP and IL-8. Dupilumab attenuates this response, potentially providing additional therapeutic benefit in asthma, especially during virus-induced exacerbations.

## Supporting information

S1 Data Raw Data Set. (PDF)
