# Supplementary material for: Dupilumab attenuates the expression of TSLP and IL-8 induced by dsRNA and IL-4/IL-13 co-stimulation in human small airway epithelial cells
Source: PLoS One. 2026 Jan 23;21(1):e0341562. doi: 10.1371/journal.pone.0341562 (PMC12829851; doi:10.1371/journal.pone.0341562)
Supplement: S1 Data — (PDF) [file pone.0341562.s001.pdf]

Raw Data HSAEC Manuscript

Figure 1 PCR TSLP

NS	ds-RNA 10 ug/mL
1	258,5
1	844,5
1	945,5

	Descriptive statistics	A<br>NS<br>Y	B<br>ds-RNA 10 ug/mL<br>Y
1	Number of values	3	3
2			
3	Minimum	1.000	258.5
4	Maximum	1.000	945.5
5	Range	0.000	687.0
6			
7	Mean	1.000	682.8
8	Std. Deviation	0.000	370.9
9	Std. Error of Mean	0.000	214.2

	Normality and Lognormality Tests<br>Tabular results	A<br>NS<br>Y	B<br>ds-RNA 10 ug/mL<br>Y
1	**Test for normal distribution**		
2	**D'Agostino & Pearson test**		
3	K2	N too small	N too small
4	P value		
5	Passed normality test (alpha=0.05)?		
6	P value summary		
7			
8	**Anderson-Darling test**		
9	A2*	N too small	N too small
10	P value		
11	Passed normality test (alpha=0.05)?		
12	P value summary		
13			
14	**Shapiro-Wilk test**		
15	W	Invalid input d	0.8575
16	P value		0.2608
17	Passed normality test (alpha=0.05)?		Yes
18	P value summary		ns
19			
20	**Kolmogorov-Smirnov test**		
21	KS distance	N too small	N too small
22	P value		
23	Passed normality test (alpha=0.05)?		
24	P value summary		
25			
26	**Number of values**	3	3

Unpaired t test<br>Tabular results	
Table Analyzed	1. NS vs ds-RNA : PCR TSLP
Column B	ds-RNA 10 ug/mL
vs.	vs.
Column A	NS
**Unpaired t test**	
P value	0.0334
P value summary	*
Significantly different (P < 0.05)?	Yes
One- or two-tailed P value?	Two-tailed
t, df	t=3.184, df=4
**How big is the difference?**	
Mean of column A	1.000
Mean of column B	682.8
Difference between means (B - A) ± SEM	681.8 ± 214.2
95% confidence interval	87.23 to 1276
R squared (eta squared)	0.7170
**F test to compare variances**	
F, DFn, Dfd	Infinity, 2, 2
P value	<0.0001
P value summary	****
Significantly different (P < 0.05)?	Yes
**Data analyzed**	
Sample size, column A	3
Sample size, column B	3

FIGURE 1 PCR IL-25

NS	ds-RNA 10 ug/mL
1	1,76
1	1,02
1	2,14

Descriptive statistics		A<br>NS	B<br>ds-RNA 10 ug/mL
		Y	Y
1	Number of values	3	3
2			
3	Minimum	1.000	1.020
4	Maximum	1.000	2.140
5	Range	0.000	1.120
6			
7	Mean	1.000	1.640
8	Std. Deviation	0.000	0.5696
9	Std. Error of Mean	0.000	0.3288

	Normality and Lognormality Tests<br>Tabular results	A<br>NS<br>Y	B<br>ds-RNA 10 ug/mL<br>Y
1	**Test for normal distribution**		
2	**D'Agostino & Pearson test**		
3	K2	N too small	N too small
4	P value		
5	Passed normality test (alpha=0.05)?		
6	P value summary		
7			
8	**Anderson-Darling test**		
9	A2*	N too small	N too small
10	P value		
11	Passed normality test (alpha=0.05)?		
12	P value summary		
13			
14	**Shapiro-Wilk test**		
15	W	Invalid input d	0.9667
16	P value		0.6496
17	Passed normality test (alpha=0.05)?		Yes
18	P value summary		ns
19			
20	**Kolmogorov-Smirnov test**		
21	KS distance	N too small	N too small
22	P value		
23	Passed normality test (alpha=0.05)?		
24	P value summary		
25			
26	**Number of values**	3	3

	Unpaired t test<br>Tabular results	
1	Table Analyzed	1. NS vs ds-RNA : PCR IL-25
2		
3	Column B	ds-RNA 10 ug/mL
4	vs.	vs.
5	Column A	NS
6		
7	**Unpaired t test**	
8	P value	0.1235
9	P value summary	ns
0	Significantly different (P < 0.05)?	No
1	One- or two-tailed P value?	Two-tailed
2	t, df	t=1.946, df=4
3		
4	**How big is the difference?**	
5	Mean of column A	1.000
6	Mean of column B	1.640
7	Difference between means (B - A) ± SEM	0.6400 ± 0.3288
8	95% confidence interval	-0.2730 to 1.553
9	R squared (eta squared)	0.4864
0		
1	**F test to compare variances**	
2	F, DFn, Dfd	Infinity, 2, 2
3	P value	<0.0001
4	P value summary	****
5	Significantly different (P < 0.05)?	Yes
6		
7	**Data analyzed**	
8	Sample size, column A	3
9	Sample size, column B	3

FIGURE 1 PCR IL-33

NS	ds-RNA 10 ug/mL
1	1,9
1	0,5
1	0,5

Descriptive statistics	A NS	B ds-RNA 10 ug/mL
	Y	Y
1 Number of values	3	3
2		
3 Minimum	1.000	0.5000
4 Maximum	1.000	1.900
5 Range	0.000	1.400
6		
7 Mean	1.000	0.9667
8 Std. Deviation	0.000	0.8083
9 Std. Error of Mean	0.000	0.4667

Normality and Lognormality Tests<br>Tabular results	A NS	B ds-RNA 10 ug/mL
	Y	Y
1 **Test for normal distribution**		
2 **D'Agostino & Pearson test**		
3 K2	N too small	N too small
4 P value		
5 Passed normality test (alpha=0.05)?		
6 P value summary		
7		
8 **Anderson-Darling test**		
9 A2*	N too small	N too small
10 P value		
11 Passed normality test (alpha=0.05)?		
12 P value summary		
13		
14 **Shapiro-Wilk test**		
15 W	Invalid input d	0.7500
16 P value		
17 Passed normality test (alpha=0.05)?		No
18 P value summary		
19		
20 **Kolmogorov-Smirnov test**		
21 KS distance	N too small	N too small
22 P value		
23 Passed normality test (alpha=0.05)?		
24 P value summary		
25		
26 **Number of values**	3	3

Unpaired t test<br>Tabular results	
1 Table Analyzed	1. NS vs ds-RNA : PCR IL-33
2	
3 Column B	ds-RNA 10 ug/mL
4 vs.	vs.
5 Column A	NS
6	
7 **Unpaired t test**	
8 P value	0.9465
9 P value summary	ns
10 Significantly different (P < 0.05)?	No
11 One- or two-tailed P value?	Two-tailed
12 t, df	t=0.07143, df=4
13	
14 **How big is the difference?**	
15 Mean of column A	1.000
16 Mean of column B	0.9667
17 Difference between means (B - A) ± SEM	-0.03333 ± 0.4667
18 95% confidence interval	-1.329 to 1.262
19 R squared (eta squared)	0.001274
20	
21 **F test to compare variances**	
22 F, DFn, Dfd	Infinity, 2, 2
23 P value	<0.0001
24 P value summary	****
25 Significantly different (P < 0.05)?	Yes
26	
27 **Data analyzed**	
28 Sample size, column A	3
29 Sample size, column B	3

FIGURE 1 PCR IL-8

NS	ds-RNA 10 ug/mL
1	11,9
1	50,53
1	45,29

Descriptive statistics	A<br>NS	B<br>ds-RNA 10 ug/mL
	Y	Y
1 Number of values	3	3
2		
3 Minimum	1.000	11.90
4 Maximum	1.000	50.53
5 Range	0.000	38.63
6		
7 Mean	1.000	35.91
8 Std. Deviation	0.000	20.95
9 Std. Error of Mean	0.000	12.10

	Normality and Lognormality Tests<br>Tabular results	A<br>NS<br>Y	B<br>ds-RNA 10 ug/mL<br>Y
1	**Test for normal distribution**		
2	**D'Agostino & Pearson test**		
3	K2	N too small	N too small
4	P value		
5	Passed normality test (alpha=0.05)?		
6	P value summary		
7			
8	**Anderson-Darling test**		
9	A2*	N too small	N too small
10	P value		
11	Passed normality test (alpha=0.05)?		
12	P value summary		
13			
14	**Shapiro-Wilk test**		
15	W	Invalid input d	0.8496
16	P value		0.2394
17	Passed normality test (alpha=0.05)?		Yes
18	P value summary		ns
19			
20	**Kolmogorov-Smirnov test**		
21	KS distance	N too small	N too small
22	P value		
23	Passed normality test (alpha=0.05)?		
24	P value summary		
25			
26	**Number of values**	3	3

	Unpaired t test<br>Tabular results	
1	Table Analyzed	1. NS vs ds-RNA : PCR IL-8
2		
3	Column B	ds-RNA 10 ug/mL
4	vs.	vs.
5	Column A	NS
6		
7	**Unpaired t test**	
8	P value	0.0448
9	P value summary	*
10	Significantly different (P < 0.05)?	Yes
11	One- or two-tailed P value?	Two-tailed
12	t, df	t=2.885, df=4
13		
14	**How big is the difference?**	
15	Mean of column A	1.000
16	Mean of column B	35.91
17	Difference between means (B - A) ± SEM	34.91 ± 12.10
18	95% confidence interval	1.316 to 68.50
19	R squared (eta squared)	0.6754
20		
21	**F test to compare variances**	
22	F, DFn, Dfd	Infinity, 2, 2
23	P value	<0.0001
24	P value summary	****
25	Significantly different (P < 0.05)?	Yes
26		
27	**Data analyzed**	
28	Sample size, column A	3
29	Sample size, column B	3

FIGURE 1 WB TSLP

NS	ds-RNA 25 ug/mL
1	1,64180663
1	1,32585669
1	1,46135841

Descriptive statistics	A	B
	NS	ds-RNA 25 ug/mL
	Y	Y
1 Number of values	3	3
2		
3 Minimum	1.000	1.326
4 Maximum	1.000	1.642
5 Range	0.000	0.3159
6		
7 Mean	1.000	1.476
8 Std. Deviation	0.000	0.1585
9 Std. Error of Mean	0.000	0.09151

Normality and Lognormality Tests<br>Tabular results	A	B
	NS	ds-RNA 25 ug/mL
	Y	Y
1 **Test for normal distribution**		
2 **D'Agostino & Pearson test**		
3 K2	N too small	N too small
4 P value		
5 Passed normality test (alpha=0.05)?		
6 P value summary		
7		
8 **Anderson-Darling test**		
9 A2*	N too small	N too small
10 P value		
11 Passed normality test (alpha=0.05)?		
12 P value summary		
13		
14 **Shapiro-Wilk test**		
15 W	Invalid input d	0.9933
16 P value		0.8435
17 Passed normality test (alpha=0.05)?		Yes
18 P value summary		ns
19		
20 **Kolmogorov-Smirnov test**		
21 KS distance	N too small	N too small
22 P value		
23 Passed normality test (alpha=0.05)?		
24 P value summary		
25		
26 **Number of values**	3	3

	Unpaired t test<br>Tabular results	
1	Table Analyzed	1. NS vs ds-RNA : Protein TSLP
2		
3	Column B	ds-RNA 25 ug/mL
4	vs.	vs.
5	Column A	NS
6		
7	**Unpaired t test**	
8	P value	0.0065
9	P value summary	**
10	Significantly different (P < 0.05)?	Yes
11	One- or two-tailed P value?	Two-tailed
12	t, df	t=5.205, df=4
13		
14	**How big is the difference?**	
15	Mean of column A	1.000
16	Mean of column B	1.476
17	Difference between means (B - A) ± SEM	0.4763 ± 0.09151
18	95% confidence interval	0.2223 to 0.7304
19	R squared (eta squared)	0.8714
20		
21	**F test to compare variances**	
22	F, DFn, Dfd	Infinity, 2, 2
23	P value	<0.0001
24	P value summary	****
25	Significantly different (P < 0.05)?	Yes
26		
27	**Data analyzed**	
28	Sample size, column A	3
29	Sample size, column B	3

FIGURE 1 WB IL-17E

NS	ds-RNA 25 ug/mL
1	1,17507363
1	0,86589893
1	1,08256238

Descriptive statistics	A<br>NS	B<br>ds-RNA 25 ug/mL
	Y	Y
Number of values	3	3
Minimum	1.000	0.8659
Maximum	1.000	1.175
Range	0.000	0.3092
Mean	1.000	1.041
Std. Deviation	0.000	0.1587
Std. Error of Mean	0.000	0.09162

	Normality and Lognormality Tests<br>Tabular results	A<br>NS<br>Y	B<br>ds-RNA 25 ug/mL<br>Y
1	**Test for normal distribution**		
2	**D'Agostino & Pearson test**		
3	K2	N too small	N too small
4	P value		
5	Passed normality test (alpha=0.05)?		
6	P value summary		
7			
8	**Anderson-Darling test**		
9	A2*	N too small	N too small
0	P value		
1	Passed normality test (alpha=0.05)?		
2	P value summary		
3			
4	**Shapiro-Wilk test**		
5	W	Invalid input d	0.9490
6	P value		0.5649
7	Passed normality test (alpha=0.05)?		Yes
8	P value summary		ns
9			
0	**Kolmogorov-Smirnov test**		
1	KS distance	N too small	N too small
2	P value		
3	Passed normality test (alpha=0.05)?		
4	P value summary		
5			
6	**Number of values**	3	3

	Unpaired t test<br>Tabular results	
1	Table Analyzed	1. NS vs ds-RNA : Protein IL-17E
2		
3	Column B	ds-RNA 25 ug/mL
4	vs.	vs.
5	Column A	NS
6		
7	**Unpaired t test**	
8	P value	0.6764
9	P value summary	ns
10	Significantly different (P < 0.05)?	No
11	One- or two-tailed P value?	Two-tailed
12	t, df	t=0.4495, df=4
13		
14	**How big is the difference?**	
15	Mean of column A	1.000
16	Mean of column B	1.041
17	Difference between means (B - A) ± SEM	0.04118 ± 0.09162
18	95% confidence interval	-0.2132 to 0.2956
19	R squared (eta squared)	0.04807
20		
21	**F test to compare variances**	
22	F, DFn, Dfd	Infinity, 2, 2
23	P value	<0.0001
24	P value summary	****
25	Significantly different (P < 0.05)?	Yes
26		
27	**Data analyzed**	
28	Sample size, column A	3
29	Sample size, column B	3

FIGURE 1 WB IL-33

NS	ds-RNA 25 ug/mL
1	1,095423007
1	0,792235544
1	1,068037949

Descriptive statistics	NS	ds-RNA 25 ug/mL
	Y	Y
Number of values	3	3
Minimum	1.000	0.7922
Maximum	1.000	1.095
Range	0.000	0.3032
Mean	1.000	0.9852
Std. Deviation	0.000	0.1677
Std. Error of Mean	0.000	0.09682

Normality and Lognormality Tests<br>Tabular results	A<br>NS	B<br>ds-RNA 25 ug/mL
	Y	Y
**Test for normal distribution**		
**D'Agostino & Pearson test**		
K2	N too small	N too small
P value		
Passed normality test (alpha=0.05)?		
P value summary		
**Anderson-Darling test**		
A2*	N too small	N too small
P value		
Passed normality test (alpha=0.05)?		
P value summary		
**Shapiro-Wilk test**		
W	Invalid input d	0.8171
P value		0.1561
Passed normality test (alpha=0.05)?		Yes
P value summary		ns
**Kolmogorov-Smirnov test**		
KS distance	N too small	N too small
P value		
Passed normality test (alpha=0.05)?		
P value summary		
**Number of values**	3	3

Unpaired t test<br>Tabular results		
1	Table Analyzed	1. NS vs ds-RNA : Protein IL-33
2		
3	Column B	ds-RNA 25 ug/mL
4	vs.	vs.
5	Column A	NS
6		
7	**Unpaired t test**	
8	P value	0.8862
9	P value summary	ns
10	Significantly different (P < 0.05)?	No
11	One- or two-tailed P value?	Two-tailed
12	t, df	t=0.1525, df=4
13		
14	**How big is the difference?**	
15	Mean of column A	1.000
16	Mean of column B	0.9852
17	Difference between means (B - A) ± SEM	-0.01477 ± 0.09682
18	95% confidence interval	-0.2836 to 0.2541
19	R squared (eta squared)	0.005782
20		
21	**F test to compare variances**	
22	F, DFn, Dfd	Infinity, 2, 2
23	P value	<0.0001
24	P value summary	****
25	Significantly different (P < 0.05)?	Yes
26		
27	**Data analyzed**	
28	Sample size, column A	3
29	Sample size, column B	3

FIGURE 1 WB IL-8

NS	ds-RNA 25 ug/mL
1	1,24789419
1	1,23522351
1	1,08824894

Descriptive statistics	A<br>NS<br>Y	B<br>ds-RNA 25 ug/mL<br>Y
Number of values	3	3
Minimum	1.000	1.088
Maximum	1.000	1.248
Range	0.000	0.1596
Mean	1.000	1.190
Std. Deviation	0.000	0.08874
Std. Error of Mean	0.000	0.05123

	Normality and Lognormality Tests<br>Tabular results	A<br>NS	B<br>ds-RNA 25 ug/mL
		Y	Y
1	**Test for normal distribution**		
2	**D'Agostino & Pearson test**		
3	K2	N too small	N too small
4	P value		
5	Passed normality test (alpha=0.05)?		
6	P value summary		
7			
8	**Anderson-Darling test**		
9	A2*	N too small	N too small
10	P value		
11	Passed normality test (alpha=0.05)?		
12	P value summary		
13			
14	**Shapiro-Wilk test**		
15	W	Invalid input d	0.8091
16	P value		0.1365
17	Passed normality test (alpha=0.05)?		Yes
18	P value summary		ns
19			
20	**Kolmogorov-Smirnov test**		
21	KS distance	N too small	N too small
22	P value		
23	Passed normality test (alpha=0.05)?		
24	P value summary		
25			
26	**Number of values**	3	3

	Unpaired t test<br>Tabular results	
1	Table Analyzed	1. NS vs ds-RNA : Protein IL-8
2		
3	Column B	ds-RNA 25 ug/mL
4	vs.	vs.
5	Column A	NS
6		
7	**Unpaired t test**	
8	P value	0.0205
9	P value summary	*
10	Significantly different (P < 0.05)?	Yes
11	One- or two-tailed P value?	Two-tailed
12	t, df	t=3.717, df=4
13		
14	**How big is the difference?**	
15	Mean of column A	1.000
16	Mean of column B	1.190
17	Difference between means (B - A) ± SEM	0.1905 ± 0.05123
18	95% confidence interval	0.04821 to 0.3327
19	R squared (eta squared)	0.7755
20		
21	**F test to compare variances**	
22	F, DFn, Dfd	Infinity, 2, 2
23	P value	<0.0001
24	P value summary	****
25	Significantly different (P < 0.05)?	Yes
26		
27	**Data analyzed**	
28	Sample size, column A	3
29	Sample size, column B	3

Figure 2 PCR TSLP

NS	IL-4	IL-5	IL-13	ds-RNA	IL-4+ds-RNA	IL-5+ds-RNA	IL-13+ds-RNA
1	2,07	2,24	2,64	5992,74	21395,21	6316,9	16180,85
1	3,34	1,2	1,71	9775,81	24525,59	8976,89	18191,79
1	0,74	1,33	2,12	9039,33	14674,26	12494,45	17782,21

Descriptive statistics	A	B	C	D	E	F	G	H
	NS	IL-4	IL-5	IL-13	ds-RNA	IL-4+ds-RNA	IL-5+ds-RNA	IL-13+ds-RNA
	Y	Y	Y	Y	Y	Y	Y	Y
1 Number of values	3	3	3	3	3	3	3	3
2								
3 Minimum	1.000	0.7400	1.200	1.710	5993	14674	6317	16181
4 Maximum	1.000	3.340	2.240	2.640	9776	24526	12494	18192
5 Range	0.000	2.600	1.040	0.9300	3783	9851	6178	2011
6								
7 Mean	1.000	2.050	1.590	2.157	8269	20198	9263	17385
8 Std. Deviation	0.000	1.300	0.5667	0.4661	2006	5034	3099	1063
9 Std. Error of Mean	0.000	0.7506	0.3272	0.2691	1158	2906	1789	613.6

Normality and Lognormality Tests<br>Tabular results	A	B	C	D	E	F	G	H
	NS	IL-4	IL-5	IL-13	ds-RNA	IL-4+ds-RNA	IL-5+ds-RNA	IL-13+ds-RNA
	Y	Y	Y	Y	Y	Y	Y	Y
1 **Test for normal distribution**								
2 **D'Agostino & Pearson test**								
3 K2	N too small	N too small	N too small	N too small	N too small	N too small	N too small	N too small
4 P value								
5 Passed normality test (alpha=0.05)?								
6 P value summary								
7								
8 **Anderson-Darling test**								
9 A2*	N too small	N too small	N too small	N too small	N too small	N too small	N too small	N too small
10 P value								
11 Passed normality test (alpha=0.05)?								
12 P value summary								
13								
14 **Shapiro-Wilk test**								
15 W	Invalid input d	0.9998	0.8421	0.9954	0.8894	0.9576	0.9936	0.8952
16 P value		0.9746	0.2196	0.8698	0.3527	0.6039	0.8473	0.3704
17 Passed normality test (alpha=0.05)?		Yes	Yes	Yes	Yes	Yes	Yes	Yes
18 P value summary		ns	ns	ns	ns	ns	ns	ns
19								
20 **Kolmogorov-Smirnov test**								
21 KS distance	N too small	N too small	N too small	N too small	N too small	N too small	N too small	N too small
22 P value								
23 Passed normality test (alpha=0.05)?								
24 P value summary								
25								
26 **Number of values**	3	3	3	3	3	3	3	3

Ordinary one-way ANOVA
ANOVA results

1	Table Analyzed	2. Th2 stimulation: PCR TSLP				
2	Data sets analyzed	A-H				
3						
4	**ANOVA summary**					
5	F	41.44				
6	P value	<0.0001				
7	P value summary	****				
8	Significant diff. among means (P < 0.05)?	Yes				
9	R squared	0.9477				
10						
11	**Brown-Forsythe test**					
12	F (DFn, DFd)	2.002 (7, 16)				
13	P value	0.1186				
14	P value summary	ns				
15	Are SDs significantly different (P < 0.05)?	No				
16						
17	**Bartlett's test**					
18	Bartlett's statistic (corrected)					
19	P value					
20	P value summary					
21	Are SDs significantly different (P < 0.05)?					
22						
23	**ANOVA table**	**SS**	**DF**	**MS**	**F (DFn, DFd)**	**P value**
24	Treatment (between columns)	1453750124	7	207678589	F (7, 16) = 41.	P<0.0001
25	Residual (within columns)	80180595	16	5011287		
26	Total	1533930718	23			
27						
28	**Data summary**					
29	Number of treatments (columns)	8				
30	Number of values (total)	24				

Ordinary one-way ANOVA
Multiple comparisons

	Tukey's multiple comparisons test	Mean Diff.	95.00% CI of diff.	Below threshold?	Summary	Adjusted P Value	
6	NS vs. IL-4	-1.050	-6329 to 6327	No	ns	>0.9999	A-B
7	NS vs. IL-5	-0.5900	-6329 to 6328	No	ns	>0.9999	A-C
8	NS vs. IL-13	-1.157	-6329 to 6327	No	ns	>0.9999	A-D
9	NS vs. ds-RNA	-8268	-14596 to -1940	Yes	**	0.0064	A-E
10	NS vs. IL-4+ds-RNA	-20197	-26525 to -13869	Yes	****	<0.0001	A-F
11	NS vs. IL-5+ds-RNA	-9262	-15590 to -2934	Yes	**	0.0022	A-G
12	NS vs. IL-13+ds-RNA	-17384	-23712 to -11056	Yes	****	<0.0001	A-H
13	IL-4 vs. IL-5	0.4600	-6328 to 6329	No	ns	>0.9999	B-C
14	IL-4 vs. IL-13	-0.1067	-6328 to 6328	No	ns	>0.9999	B-D
15	IL-4 vs. ds-RNA	-8267	-14595 to -1939	Yes	**	0.0065	B-E
16	IL-4 vs. IL-4+ds-RNA	-20196	-26524 to -13868	Yes	****	<0.0001	B-F
17	IL-4 vs. IL-5+ds-RNA	-9261	-15589 to -2933	Yes	**	0.0022	B-G
18	IL-4 vs. IL-13+ds-RNA	-17383	-23711 to -11055	Yes	****	<0.0001	B-H
19	IL-5 vs. IL-13	-0.5667	-6329 to 6328	No	ns	>0.9999	C-D
20	IL-5 vs. ds-RNA	-8268	-14596 to -1940	Yes	**	0.0065	C-E
21	IL-5 vs. IL-4+ds-RNA	-20197	-26525 to -13869	Yes	****	<0.0001	C-F
22	IL-5 vs. IL-5+ds-RNA	-9261	-15589 to -2933	Yes	**	0.0022	C-G
23	IL-5 vs. IL-13+ds-RNA	-17383	-23711 to -11055	Yes	****	<0.0001	C-H
24	IL-13 vs. ds-RNA	-8267	-14595 to -1939	Yes	**	0.0065	D-E
25	IL-13 vs. IL-4+ds-RNA	-20196	-26524 to -13868	Yes	****	<0.0001	D-F
26	IL-13 vs. IL-5+ds-RNA	-9261	-15589 to -2932	Yes	**	0.0022	D-G
27	IL-13 vs. IL-13+ds-RNA	-17383	-23711 to -11055	Yes	****	<0.0001	D-H
28	ds-RNA vs. IL-4+ds-RNA	-11929	-18257 to -5601	Yes	***	0.0001	E-F
29	ds-RNA vs. IL-5+ds-RNA	-993.5	-7322 to 5335	No	ns	0.9991	E-G
30	ds-RNA vs. IL-13+ds-RNA	-9116	-15444 to -2788	Yes	**	0.0026	E-H
31	IL-4+ds-RNA vs. IL-5+ds-RNA	10936	4607 to 17264	Yes	***	0.0004	F-G
32	IL-4+ds-RNA vs. IL-13+ds-RNA	2813	-3515 to 9142	No	ns	0.7768	F-H
33	IL-5+ds-RNA vs. IL-13+ds-RNA	-8122	-14450 to -1794	Yes	**	0.0075	G-H

Figure 2 PCR IL-25

NS	IL-4	IL-5	IL-13	ds-RNA	IL-4+ds-RNA	IL-5+ds-RNA	IL-13+ds-RNA
1	1,43	2,05	1,38	2,57	2,16	2,6	4,64
1	2,25	1,34	1,49	4,58	6,23	5,53	2,91
1	1,68	0,99	0,92	2,6	4,47	3,63	1,34
1	1,54	3,83	3,98	1,08	1,94	1,85	2,31
1	1,29	1,39	2,12	3,99	2,52	1,8	2,48

Descriptive statistics

		A	B	C	D	E	F	G	H
		NS	IL-4	IL-5	IL-13	ds-RNA	IL-4+ds-RNA	IL-5+ds-RNA	IL-13+ds-RNA
		Y	Y	Y	Y	Y	Y	Y	Y
1	Number of values	5	5	5	5	5	5	5	5
2									
3	Minimum	1.000	1.290	0.9900	0.9200	1.080	1.940	1.800	1.340
4	Maximum	1.000	2.250	3.830	3.980	4.580	6.230	5.530	4.640
5	Range	0.000	0.9600	2.840	3.060	3.500	4.290	3.730	3.300
6									
7	Mean	1.000	1.638	1.920	1.978	2.964	3.464	3.082	2.736
8	Std. Deviation	0.000	0.3709	1.134	1.198	1.369	1.842	1.556	1.209
9	Std. Error of Mean	0.000	0.1659	0.5073	0.5359	0.6124	0.8239	0.6959	0.5409

Normality and Lognormality Tests<br>Tabular results		A<br>NS	B<br>IL-4	C<br>IL-5	D<br>IL-13	E<br>ds-RNA	F<br>IL-4+ds-RNA	G<br>IL-5+ds-RNA	H<br>IL-13+ds-RNA
		Y	Y	Y	Y	Y	Y	Y	Y
1	**Test for normal distribution**								
2	**D'Agostino & Pearson test**								
3	K2	N too small	N too small	N too small	N too small	N too small	N too small	N too small	N too small
4	P value								
5	Passed normality test (alpha=0.05)?								
6	P value summary								
7									
8	**Anderson-Darling test**								
9	A2*	N too small	N too small	N too small	N too small	N too small	N too small	N too small	N too small
10	P value								
11	Passed normality test (alpha=0.05)?								
12	P value summary								
13									
14	**Shapiro-Wilk test**								
15	W	Invalid input d	0.8864	0.8236	0.8523	0.9496	0.8559	0.8734	0.9343
16	P value		0.3392	0.1244	0.2017	0.7345	0.2138	0.2806	0.6259
17	Passed normality test (alpha=0.05)?		Yes	Yes	Yes	Yes	Yes	Yes	Yes
18	P value summary		ns	ns	ns	ns	ns	ns	ns
19									
20	**Kolmogorov-Smirnov test**								
21	KS distance	1.000	0.2549	0.2798	0.2581	0.2048	0.2958	0.2216	0.2428
22	P value	<0.0001	>0.1000	>0.1000	>0.1000	>0.1000	>0.1000	>0.1000	>0.1000
23	Passed normality test (alpha=0.05)?	No	Yes	Yes	Yes	Yes	Yes	Yes	Yes
24	P value summary	****	ns	ns	ns	ns	ns	ns	ns
25									
26	**Number of values**	5	5	5	5	5	5	5	5

	Ordinary one-way ANOVA<br>ANOVA results					
1	Table Analyzed	2. Th2 stimulation: PCR IL-25				
2	Data sets analyzed	A-H				
3						
4	**ANOVA summary**					
5	F	2.357				
6	P value	0.0463				
7	P value summary	*				
8	Significant diff. among means (P < 0.05)?	Yes				
9	R squared	0.3402				
10						
11	**Brown-Forsythe test**					
12	F (DFn, DFd)	1.047 (7, 32)				
13	P value	0.4192				
14	P value summary	ns				
15	Are SDs significantly different (P < 0.05)?	No				
16						
17	**Bartlett's test**					
18	Bartlett's statistic (corrected)					
19	P value					
20	P value summary					
21	Are SDs significantly different (P < 0.05)?					
22						
23	**ANOVA table**	**SS**	**DF**	**MS**	**F (DFn, DFd)**	**P value**
24	Treatment (between columns)	24.78	7	3.540	F (7, 32) = 2.357	P=0.0463
25	Residual (within columns)	48.06	32	1.502		
26	Total	72.83	39			
27						
28	**Data summary**					
29	Number of treatments (columns)	8				
30	Number of values (total)	40				

Ordinary one-way ANOVA
Multiple comparisons

	Tukey's multiple comparisons test	Mean Diff.	95.00% CI of diff.	Below threshold?	Summary	Adjusted P Value	
6	NS vs. IL-4	-0.6380	-3.149 to 1.873	No	ns	0.9904	A-B
7	NS vs. IL-5	-0.9200	-3.431 to 1.591	No	ns	0.9298	A-C
8	NS vs. IL-13	-0.9780	-3.489 to 1.533	No	ns	0.9060	A-D
9	NS vs. ds-RNA	-1.964	-4.475 to 0.5466	No	ns	0.2179	A-E
10	NS vs. IL-4+ds-RNA	-2.464	-4.975 to 0.04664	No	ns	0.0574	A-F
11	NS vs. IL-5+ds-RNA	-2.082	-4.593 to 0.4286	No	ns	0.1637	A-G
12	NS vs. IL-13+ds-RNA	-1.736	-4.247 to 0.7746	No	ns	0.3563	A-H
13	IL-4 vs. IL-5	-0.2820	-2.793 to 2.229	No	ns	>0.9999	B-C
14	IL-4 vs. IL-13	-0.3400	-2.851 to 2.171	No	ns	0.9998	B-D
15	IL-4 vs. ds-RNA	-1.326	-3.837 to 1.185	No	ns	0.6805	B-E
16	IL-4 vs. IL-4+ds-RNA	-1.826	-4.337 to 0.6846	No	ns	0.2964	B-F
17	IL-4 vs. IL-5+ds-RNA	-1.444	-3.955 to 1.067	No	ns	0.5843	B-G
18	IL-4 vs. IL-13+ds-RNA	-1.098	-3.609 to 1.413	No	ns	0.8429	B-H
19	IL-5 vs. IL-13	-0.05800	-2.569 to 2.453	No	ns	>0.9999	C-D
20	IL-5 vs. ds-RNA	-1.044	-3.555 to 1.467	No	ns	0.8736	C-E
21	IL-5 vs. IL-4+ds-RNA	-1.544	-4.055 to 0.9666	No	ns	0.5024	C-F
22	IL-5 vs. IL-5+ds-RNA	-1.162	-3.673 to 1.349	No	ns	0.8022	C-G
23	IL-5 vs. IL-13+ds-RNA	-0.8160	-3.327 to 1.695	No	ns	0.9619	C-H
24	IL-13 vs. ds-RNA	-0.9860	-3.497 to 1.525	No	ns	0.9024	D-E
25	IL-13 vs. IL-4+ds-RNA	-1.486	-3.997 to 1.025	No	ns	0.5498	D-F
26	IL-13 vs. IL-5+ds-RNA	-1.104	-3.615 to 1.407	No	ns	0.8393	D-G
27	IL-13 vs. IL-13+ds-RNA	-0.7580	-3.269 to 1.753	No	ns	0.9744	D-H
28	ds-RNA vs. IL-4+ds-RNA	-0.5000	-3.011 to 2.011	No	ns	0.9978	E-F
29	ds-RNA vs. IL-5+ds-RNA	-0.1180	-2.629 to 2.393	No	ns	>0.9999	E-G
30	ds-RNA vs. IL-13+ds-RNA	0.2280	-2.283 to 2.739	No	ns	>0.9999	E-H
31	IL-4+ds-RNA vs. IL-5+ds-RNA	0.3820	-2.129 to 2.893	No	ns	0.9996	F-G
32	IL-4+ds-RNA vs. IL-13+ds-RNA	0.7280	-1.783 to 3.239	No	ns	0.9795	F-H
33	IL-5+ds-RNA vs. IL-13+ds-RNA	0.3460	-2.165 to 2.857	No	ns	0.9998	G-H

Figure 2 PCR IL-33

NS	IL-4	IL-5	IL-13	ds-RNA	IL-4+ds-RNA	IL-5+ds-RNA	IL-13+ds-RNA
1	1,02	1,31	0,88	0,84	0,86	1	0,83
1	1,17	0,78	1,16	0,95	0,58	1,13	1,18
1	1,24	1,04	2,02	1,85	1,67	1,75	1,23
1	0,75	1,49	0,99	0,97	0,52	0,98	0,62
1	0,54	1,22	0,75	1,44	0,46	0,69	0,69

Descriptive statistics		A	B	C	D	E	F	G	H
		NS	IL-4	IL-5	IL-13	ds-RNA	IL-4+ds-RNA	IL-5+ds-RNA	IL-13+ds-RNA
		Y	Y	Y	Y	Y	Y	Y	Y
1	Number of values	5	5	5	5	5	5	5	5
2									
3	Minimum	1.000	0.5400	0.7800	0.7500	0.8400	0.4600	0.6900	0.6200
4	Maximum	1.000	1.240	1.490	2.020	1.850	1.670	1.750	1.230
5	Range	0.000	0.7000	0.7100	1.270	1.010	1.210	1.060	0.6100
6									
7	Mean	1.000	0.9440	1.168	1.160	1.210	0.8180	1.110	0.9100
8	Std. Deviation	0.000	0.2938	0.2709	0.5037	0.4256	0.5003	0.3922	0.2803
9	Std. Error of Mean	0.000	0.1314	0.1211	0.2253	0.1903	0.2237	0.1754	0.1253

Normality and Lognormality Tests<br>Tabular results		A	B	C	D	E	F	G	H
		NS	IL-4	IL-5	IL-13	ds-RNA	IL-4+ds-RNA	IL-5+ds-RNA	IL-13+ds-RNA
		Y	Y	Y	Y	Y	Y	Y	Y
1	**Test for normal distribution**								
2	**D'Agostino & Pearson test**								
3	K2	N too small	N too small	N too small	N too small	N too small	N too small	N too small	N too small
4	P value								
5	Passed normality test (alpha=0.05)?								
6	P value summary								
7									
8	**Anderson-Darling test**								
9	A2*	N too small	N too small	N too small	N too small	N too small	N too small	N too small	N too small
10	P value								
11	Passed normality test (alpha=0.05)?								
12	P value summary								
13									
14	**Shapiro-Wilk test**								
15	W	Invalid input d	0.9297	0.9828	0.8189	0.8603	0.7845	0.8916	0.8729
16	P value		0.5946	0.9491	0.1144	0.2294	0.0602	0.3650	0.2783
17	Passed normality test (alpha=0.05)?		Yes	Yes	Yes	Yes	Yes	Yes	Yes
18	P value summary		ns	ns	ns	ns	ns	ns	ns
19									
20	**Kolmogorov-Smirnov test**								
21	KS distance	1.000	0.2021	0.1761	0.3000	0.3136	0.2829	0.2797	0.2323
22	P value	<0.0001	>0.1000	>0.1000	>0.1000	>0.1000	>0.1000	>0.1000	>0.1000
23	Passed normality test (alpha=0.05)?	No	Yes	Yes	Yes	Yes	Yes	Yes	Yes
24	P value summary	****	ns	ns	ns	ns	ns	ns	ns
25									
26	**Number of values**	5	5	5	5	5	5	5	5

Ordinary one-way ANOVA
ANOVA results

1	Table Analyzed	2. Th2 stimulation: PCR IL-33				
2	Data sets analyzed	A-H				
3						
4	**ANOVA summary**					
5	F	0.7510				
6	P value	0.6314				
7	P value summary	ns				
8	Significant diff. among means (P < 0.05)?	No				
9	R squared	0.1411				
10						
11	**Brown-Forsythe test**					
12	F (DFn, DFd)	0.5927 (7, 32)				
13	P value	0.7569				
14	P value summary	ns				
15	Are SDs significantly different (P < 0.05)?	No				
16						
17	**Bartlett's test**					
18	Bartlett's statistic (corrected)					
19	P value					
20	P value summary					
21	Are SDs significantly different (P < 0.05)?					
22						
23	**ANOVA table**	**SS**	**DF**	**MS**	**F (DFn, DFd)**	**P value**
24	Treatment (between columns)	0.7079	7	0.1011	F (7, 32) = 0.7510	P=0.6314
25	Residual (within columns)	4.309	32	0.1347		
26	Total	5.017	39			
27						
28	**Data summary**					
29	Number of treatments (columns)	8				
30	Number of values (total)	40				

Ordinary one-way ANOVA
Multiple comparisons

Tukey's multiple comparisons test	95.00% CI of diff.	Below threshold?	Summary	Adjusted P Value	
NS vs. IL-4	-0.6958 to 0.8078	No	ns	>0.9999	A-B
NS vs. IL-5	-0.9198 to 0.5838	No	ns	0.9956	A-C
NS vs. IL-13	-0.9118 to 0.5918	No	ns	0.9967	A-D
NS vs. ds-RNA	-0.9618 to 0.5418	No	ns	0.9834	A-E
NS vs. IL-4+ds-RNA	-0.5698 to 0.9338	No	ns	0.9928	A-F
NS vs. IL-5+ds-RNA	-0.8618 to 0.6418	No	ns	0.9997	A-G
NS vs. IL-13+ds-RNA	-0.6618 to 0.8418	No	ns	>0.9999	A-H
IL-4 vs. IL-5	-0.9758 to 0.5278	No	ns	0.9762	B-C
IL-4 vs. IL-13	-0.9678 to 0.5358	No	ns	0.9806	B-D
IL-4 vs. ds-RNA	-1.018 to 0.4858	No	ns	0.9410	B-E
IL-4 vs. IL-4+ds-RNA	-0.6258 to 0.8778	No	ns	0.9993	B-F
IL-4 vs. IL-5+ds-RNA	-0.9178 to 0.5858	No	ns	0.9959	B-G
IL-4 vs. IL-13+ds-RNA	-0.7178 to 0.7858	No	ns	>0.9999	B-H
IL-5 vs. IL-13	-0.7438 to 0.7598	No	ns	>0.9999	C-D
IL-5 vs. ds-RNA	-0.7938 to 0.7098	No	ns	>0.9999	C-E
IL-5 vs. IL-4+ds-RNA	-0.4018 to 1.102	No	ns	0.7976	C-F
IL-5 vs. IL-5+ds-RNA	-0.6938 to 0.8098	No	ns	>0.9999	C-G
IL-5 vs. IL-13+ds-RNA	-0.4938 to 1.010	No	ns	0.9495	C-H
IL-13 vs. ds-RNA	-0.8018 to 0.7018	No	ns	>0.9999	D-E
IL-13 vs. IL-4+ds-RNA	-0.4098 to 1.094	No	ns	0.8153	D-F
IL-13 vs. IL-5+ds-RNA	-0.7018 to 0.8018	No	ns	>0.9999	D-G
IL-13 vs. IL-13+ds-RNA	-0.5018 to 1.002	No	ns	0.9571	D-H
ds-RNA vs. IL-4+ds-RNA	-0.3598 to 1.144	No	ns	0.6939	E-F
ds-RNA vs. IL-5+ds-RNA	-0.6518 to 0.8518	No	ns	0.9998	E-G
ds-RNA vs. IL-13+ds-RNA	-0.4518 to 1.052	No	ns	0.8950	E-H
IL-4+ds-RNA vs. IL-5+ds-RNA	-1.044 to 0.4598	No	ns	0.9073	F-G
IL-4+ds-RNA vs. IL-13+ds-RNA	-0.8438 to 0.6598	No	ns	>0.9999	F-H
IL-5+ds-RNA vs. IL-13+ds-RNA	-0.5518 to 0.9518	No	ns	0.9875	G-H

Figure 2 PCR IL-8

NS	IL-4	IL-5	IL-13	ds-RNA	IL-4+ds-RNA	IL-5+ds-RNA	IL-13+ds-RNA
1	1,34	0,83	1,38	15,2	30,76	19,45	31,04
1	1,33	0,9	1,82	17,07	23,75	16,71	27,99
1	1,46	1,08	1,54	13,82	26,1	17,72	20,08

Descriptive statistics	NS	IL-4	IL-5	IL-13	ds-RNA	IL-4+ds-RNA	IL-5+ds-RNA	IL-13+ds-RNA
	Y	Y	Y	Y	Y	Y	Y	Y
Number of values	3	3	3	3	3	3	3	3
Minimum	1.000	1.330	0.8300	1.380	13.82	23.75	16.71	20.08
Maximum	1.000	1.460	1.080	1.820	17.07	30.76	19.45	31.04
Range	0.000	0.1300	0.2500	0.4400	3.250	7.010	2.740	10.96
Mean	1.000	1.377	0.9367	1.580	15.36	26.87	17.96	26.37
Std. Deviation	0.000	0.07234	0.1290	0.2227	1.631	3.568	1.386	5.657
Std. Error of Mean	0.000	0.04177	0.07446	0.1286	0.9417	2.060	0.8000	3.266

Normality and Lognormality Tests<br>Tabular results	A	B	C	D	E	F	G	H
	NS	IL-4	IL-5	IL-13	ds-RNA	IL-4+ds-RNA	IL-5+ds-RNA	IL-13+ds-RNA
	Y	Y	Y	Y	Y	Y	Y	Y
1 **Test for normal distribution**								
2 **D'Agostino & Pearson test**								
3 K2	N too small	N too small	N too small	N too small	N too small	N too small	N too small	N too small
4 P value								
5 Passed normality test (alpha=0.05)?								
6 P value summary								
7								
8 **Anderson-Darling test**								
9 A2*	N too small	N too small	N too small	N too small	N too small	N too small	N too small	N too small
10 P value								
11 Passed normality test (alpha=0.05)?								
12 P value summary								
13								
14 **Shapiro-Wilk test**								
15 W	Invalid input d	0.8073	0.9394	0.9758	0.9925	0.9651	0.9775	0.9385
16 P value		0.1321	0.5249	0.7017	0.8342	0.6409	0.7124	0.5213
17 Passed normality test (alpha=0.05)?		Yes	Yes	Yes	Yes	Yes	Yes	Yes
18 P value summary		ns	ns	ns	ns	ns	ns	ns
19								
20 **Kolmogorov-Smirnov test**								
21 KS distance	N too small	N too small	N too small	N too small	N too small	N too small	N too small	N too small
22 P value								
23 Passed normality test (alpha=0.05)?								
24 P value summary								
25								
26 **Number of values**	3	3	3	3	3	3	3	3

Ordinary one-way ANOVA
ANOVA results

Table Analyzed	2. Th2 stimulation: PCR IL-8				
Data sets analyzed	A-H				
**ANOVA summary**					
F	65.04				
P value	<0.0001				
P value summary	****				
Significant diff. among means (P < 0.05)?	Yes				
R squared	0.9660				
**Brown-Forsythe test**					
F (DFn, DFd)	1.830 (7, 16)				
P value	0.1499				
P value summary	ns				
Are SDs significantly different (P < 0.05)?	No				
**Bartlett's test**					
Bartlett's statistic (corrected)					
P value					
P value summary					
Are SDs significantly different (P < 0.05)?					
**ANOVA table**	**SS**	**DF**	**MS**	**F (DFn, DFd)**	**P value**
Treatment (between columns)	2810	7	401.4	F (7, 16) = 65.04	P<0.0001
Residual (within columns)	98.76	16	6.173		
Total	2909	23			
**Data summary**					
Number of treatments (columns)	8				
Number of values (total)	24				

Ordinary one-way ANOVA
Multiple comparisons

Tukey's multiple comparisons test	Mean Diff.	95.00% CI of diff.	Below threshold?	Summary	Adjusted P Value	
NS vs. IL-4	-0.3767	-7.400 to 6.647	No	ns	>0.9999	A-B
NS vs. IL-5	0.06333	-6.960 to 7.087	No	ns	>0.9999	A-C
NS vs. IL-13	-0.5800	-7.603 to 6.443	No	ns	>0.9999	A-D
NS vs. ds-RNA	-14.36	-21.39 to -7.340	Yes	****	<0.0001	A-E
NS vs. IL-4+ds-RNA	-25.87	-32.89 to -18.85	Yes	****	<0.0001	A-F
NS vs. IL-5+ds-RNA	-16.96	-23.98 to -9.937	Yes	****	<0.0001	A-G
NS vs. IL-13+ds-RNA	-25.37	-32.39 to -18.35	Yes	****	<0.0001	A-H
IL-4 vs. IL-5	0.4400	-6.583 to 7.463	No	ns	>0.9999	B-C
IL-4 vs. IL-13	-0.2033	-7.227 to 6.820	No	ns	>0.9999	B-D
IL-4 vs. ds-RNA	-13.99	-21.01 to -6.963	Yes	****	<0.0001	B-E
IL-4 vs. IL-4+ds-RNA	-25.49	-32.52 to -18.47	Yes	****	<0.0001	B-F
IL-4 vs. IL-5+ds-RNA	-16.58	-23.61 to -9.560	Yes	****	<0.0001	B-G
IL-4 vs. IL-13+ds-RNA	-24.99	-32.02 to -17.97	Yes	****	<0.0001	B-H
IL-5 vs. IL-13	-0.6433	-7.667 to 6.380	No	ns	>0.9999	C-D
IL-5 vs. ds-RNA	-14.43	-21.45 to -7.403	Yes	****	<0.0001	C-E
IL-5 vs. IL-4+ds-RNA	-25.93	-32.96 to -18.91	Yes	****	<0.0001	C-F
IL-5 vs. IL-5+ds-RNA	-17.02	-24.05 to -10.00	Yes	****	<0.0001	C-G
IL-5 vs. IL-13+ds-RNA	-25.43	-32.46 to -18.41	Yes	****	<0.0001	C-H
IL-13 vs. ds-RNA	-13.78	-20.81 to -6.760	Yes	****	<0.0001	D-E
IL-13 vs. IL-4+ds-RNA	-25.29	-32.31 to -18.27	Yes	****	<0.0001	D-F
IL-13 vs. IL-5+ds-RNA	-16.38	-23.40 to -9.357	Yes	****	<0.0001	D-G
IL-13 vs. IL-13+ds-RNA	-24.79	-31.81 to -17.77	Yes	****	<0.0001	D-H
ds-RNA vs. IL-4+ds-RNA	-11.51	-18.53 to -4.483	Yes	***	0.0007	E-F
ds-RNA vs. IL-5+ds-RNA	-2.597	-9.620 to 4.427	No	ns	0.8936	E-G
ds-RNA vs. IL-13+ds-RNA	-11.01	-18.03 to -3.983	Yes	**	0.0011	E-H
IL-4+ds-RNA vs. IL-5+ds-RNA	8.910	1.887 to 15.93	Yes	**	0.0083	F-G
IL-4+ds-RNA vs. IL-13+ds-RNA	0.5000	-6.523 to 7.523	No	ns	>0.9999	F-H
IL-5+ds-RNA vs. IL-13+ds-RNA	-8.410	-15.43 to -1.387	Yes	*	0.0135	G-H

Figure 2 WB TSLP

NS	ds-RNA	IL-4+ds-RNA	IL-13+ds-RNA
1	1,23826924	1,44252603	2,07117137
1	1,15627759	1,45359513	1,85803494
1	1,24884461	1,52374554	1,91401323

Descriptive statistics	NS	ds-RNA	IL-4+ds-RNA	IL-13+ds-RNA
	Y	Y	Y	Y
Number of values	3	3	3	3
Minimum	1.000	1.156	1.443	1.858
Maximum	1.000	1.249	1.524	2.071
Range	0.000	0.09257	0.08122	0.2131
Mean	1.000	1.214	1.473	1.948
Std. Deviation	0.000	0.05067	0.04405	0.1105
Std. Error of Mean	0.000	0.02925	0.02543	0.06380

	Normality and Lognormality Tests<br>Tabular results	A<br>NS	B<br>ds-RNA	C<br>IL-4+ds-RNA	D<br>IL-13+ds-RNA
		Y	Y	Y	Y
1	**Test for normal distribution**				
2	**D'Agostino & Pearson test**				
3	K2	N too small	N too small	N too small	N too small
4	P value				
5	Passed normality test (alpha=0.05)?				
6	P value summary				
7					
8	**Anderson-Darling test**				
9	A2*	N too small	N too small	N too small	N too small
10	P value				
11	Passed normality test (alpha=0.05)?				
12	P value summary				
13					
14	**Shapiro-Wilk test**				
15	W	Invalid input d	0.8344	0.8501	0.9301
16	P value		0.1997	0.2406	0.4891
17	Passed normality test (alpha=0.05)?		Yes	Yes	Yes
18	P value summary		ns	ns	ns
19					
20	**Kolmogorov-Smirnov test**				
21	KS distance	N too small	N too small	N too small	N too small
22	P value				
23	Passed normality test (alpha=0.05)?				
24	P value summary				
25					
26	**Number of values**	3	3	3	3

	Ordinary one-way ANOVA<br>ANOVA results					
1	Table Analyzed	2. Th2 stimulation: Protein TSLP 4 groups				
2	Data sets analyzed	A-D				
3						
4	**ANOVA summary**					
5	F	119.5				
6	P value	<0.0001				
7	P value summary	****				
8	Significant diff. among means (P < 0.05)?	Yes				
9	R squared	0.9782				
10						
11	**Brown-Forsythe test**					
12	F (DFn, DFd)	1.055 (3, 8)				
13	P value	0.4200				
14	P value summary	ns				
15	Are SDs significantly different (P < 0.05)?	No				
16						
17	**Bartlett's test**					
18	Bartlett's statistic (corrected)					
19	P value					
20	P value summary					
21	Are SDs significantly different (P < 0.05)?					
22						
23	**ANOVA table**	**SS**	**DF**	**MS**	**F (DFn, DFd)**	**P value**
24	Treatment (between columns)	1.498	3	0.4995	F (3, 8) = 119.5	P<0.0001
25	Residual (within columns)	0.03343	8	0.004179		
26	Total	1.532	11			
27						
28	**Data summary**					
29	Number of treatments (columns)	4				
30	Number of values (total)	12				

Ordinary one-way ANOVA Multiple comparisons									
1	Number of families	1							
2	Number of comparisons per family	6							
3	Alpha	0.05							
4									
5	**Tukey's multiple comparisons test**	**Mean Diff.**	**95.00% CI of diff.**	**Below threshold?**	**Summary**	**Adjusted P Value**			
6	NS vs. ds-RNA	-0.2145	-0.3835 to -0.04543	Yes	*	0.0153	A-B		
7	NS vs. IL-4+ds-RNA	-0.4733	-0.6423 to -0.3043	Yes	****	<0.0001	A-C		
8	NS vs. IL-13+ds-RNA	-0.9477	-1.117 to -0.7787	Yes	****	<0.0001	A-D		
9	ds-RNA vs. IL-4+ds-RNA	-0.2588	-0.4279 to -0.08979	Yes	**	0.0052	B-C		
10	ds-RNA vs. IL-13+ds-RNA	-0.7333	-0.9023 to -0.5642	Yes	****	<0.0001	B-D		
11	IL-4+ds-RNA vs. IL-13+ds-RNA	-0.4745	-0.6435 to -0.3054	Yes	****	<0.0001	C-D		
12									
13	**Test details**	**Mean 1**	**Mean 2**	**Mean Diff.**	**SE of diff.**	**n1**	**n2**	**q**	**DF**
14	NS vs. ds-RNA	1.000	1.214	-0.2145	0.05278	3	3	5.746	8
15	NS vs. IL-4+ds-RNA	1.000	1.473	-0.4733	0.05278	3	3	12.68	8
16	NS vs. IL-13+ds-RNA	1.000	1.948	-0.9477	0.05278	3	3	25.39	8
17	ds-RNA vs. IL-4+ds-RNA	1.214	1.473	-0.2588	0.05278	3	3	6.935	8
18	ds-RNA vs. IL-13+ds-RNA	1.214	1.948	-0.7333	0.05278	3	3	19.65	8
19	IL-4+ds-RNA vs. IL-13+ds-RNA	1.473	1.948	-0.4745	0.05278	3	3	12.71	8
20									
21	**Compact letter display**								
22	IL-13+ds-RNA	A							
23	IL-4+ds-RNA	B							
24	ds-RNA	C							
25	NS	D							

Figure 2 WB IL-8

NS	ds-RNA	IL-4+ds-RNA	IL-13+ds-RNA
1	1,169920621	1,48834283	1,310231912
1	1,135088476	1,317425114	1,324417117
1	1,1557572	1,340703358	1,298854757

	Descriptive statistics	NS	ds-RNA	IL-4+ds-RNA	IL-13+ds-RNA
		Y	Y	Y	Y
1	Number of values	3	3	3	3
2					
3	Minimum	1.000	1.135	1.317	1.299
4	Maximum	1.000	1.170	1.488	1.324
5	Range	0.000	0.03483	0.1709	0.02556
6					
7	Mean	1.000	1.154	1.382	1.311
8	Std. Deviation	0.000	0.01752	0.09269	0.01281
9	Std. Error of Mean	0.000	0.01011	0.05352	0.007394

Normality and Lognormality Tests<br>Tabular results	A	B	C	D
	NS	ds-RNA	IL-4+ds-RNA	IL-13+ds-RNA
	Y	Y	Y	Y
**Test for normal distribution**				
**D'Agostino & Pearson test**				
K2	N too small	N too small	N too small	N too small
P value				
Passed normality test (alpha=0.05)?				
P value summary				
**Anderson-Darling test**				
A2*	N too small	N too small	N too small	N too small
P value				
Passed normality test (alpha=0.05)?				
P value summary				
**Shapiro-Wilk test**				
W	Invalid input d	0.9885	0.8500	0.9960
P value		0.7949	0.2404	0.8790
Passed normality test (alpha=0.05)?		Yes	Yes	Yes
P value summary		ns	ns	ns
**Kolmogorov-Smirnov test**				
KS distance	N too small	N too small	N too small	N too small
P value				
Passed normality test (alpha=0.05)?				
P value summary				
**Number of values**	3	3	3	3

	Ordinary one-way ANOVA<br>ANOVA results					
1	Table Analyzed	2. Th2 stimulation: Protein IL-8 4 groups				
2	Data sets analyzed	A-D				
3						
4	**ANOVA summary**					
5	F	38.46				
6	P value	<0.0001				
7	P value summary	****				
8	Significant diff. among means (P < 0.05)?	Yes				
9	R squared	0.9352				
10						
11	**Brown-Forsythe test**					
12	F (DFn, DFd)	1.216 (3, 8)				
13	P value	0.3648				
14	P value summary	ns				
15	Are SDs significantly different (P < 0.05)?	No				
16						
17	**Bartlett's test**					
18	Bartlett's statistic (corrected)					
19	P value					
20	P value summary					
21	Are SDs significantly different (P < 0.05)?					
22						
23	**ANOVA table**	**SS**	**DF**	**MS**	**F (DFn, DFd)**	**P value**
24	Treatment (between columns)	0.2614	3	0.08714	F (3, 8) = 38.46	P<0.0001
25	Residual (within columns)	0.01813	8	0.002266		
26	Total	0.2796	11			
27						
28	**Data summary**					
29	Number of treatments (columns)	4				
30	Number of values (total)	12				

	Ordinary one-way ANOVA<br>Multiple comparisons								
1	Number of families	1							
2	Number of comparisons per family	6							
3	Alpha	0.05							
4									
5	**Tukey's multiple comparisons test**	**Mean Diff.**	**95.00% CI of diff.**	**Below threshold?**	**Summary**	**Adjusted P Value**			
6	NS vs. ds-RNA	-0.1536	-0.2780 to -0.02913	Yes	*	0.0178	A-B		
7	NS vs. IL-4+ds-RNA	-0.3822	-0.5066 to -0.2577	Yes	****	<0.0001	A-C		
8	NS vs. IL-13+ds-RNA	-0.3112	-0.4356 to -0.1867	Yes	***	0.0002	A-D		
9	ds-RNA vs. IL-4+ds-RNA	-0.2286	-0.3530 to -0.1041	Yes	**	0.0017	B-C		
10	ds-RNA vs. IL-13+ds-RNA	-0.1576	-0.2820 to -0.03312	Yes	*	0.0155	B-D		
11	IL-4+ds-RNA vs. IL-13+ds-RNA	0.07099	-0.05347 to 0.1954	No	ns	0.3288	C-D		
12									
13	**Test details**	**Mean 1**	**Mean 2**	**Mean Diff.**	**SE of diff.**	**n1**	**n2**	**q**	**DF**
14	NS vs. ds-RNA	1.000	1.154	-0.1536	0.03886	3	3	5.589	8
15	NS vs. IL-4+ds-RNA	1.000	1.382	-0.3822	0.03886	3	3	13.91	8
16	NS vs. IL-13+ds-RNA	1.000	1.311	-0.3112	0.03886	3	3	11.32	8
17	ds-RNA vs. IL-4+ds-RNA	1.154	1.382	-0.2286	0.03886	3	3	8.317	8
18	ds-RNA vs. IL-13+ds-RNA	1.154	1.311	-0.1576	0.03886	3	3	5.734	8
19	IL-4+ds-RNA vs. IL-13+ds-RNA	1.382	1.311	0.07099	0.03886	3	3	2.583	8
20									
21	**Compact letter display**								
22	IL-4+ds-RNA	A							
23	IL-13+ds-RNA	A							
24	ds-RNA	B							
25	NS	C							

Figure 3 PCR TSLP

NS	CXCL-8	LTB-4	TNF-a	ds-RNA	CXCL-8+ds-RNA	LTB-4+ds-RNA	TNF-a+ds-RNA
1	0,62	0,93	0,9	1402,71	1240,75	1146,48	333,61
1	0,79	0,86	0,54	2509,18	2227,18	2262,97	537,83
1	0,49	0,47	0,49	1738,95	1713,82	1633,78	355,32

	Descriptive statistics	A<br>NS	B<br>CXCL-8	C<br>LTB-4	D<br>TNF-a	E<br>ds-RNA	F<br>CXCL-8+ds-RNA	G<br>LTB-4+ds-RNA	H<br>TNF-a+ds-RNA
		Y	Y	Y	Y	Y	Y	Y	Y
1	Number of values	3	3	3	3	3	3	3	3
2									
3	Minimum	1.000	0.4900	0.4700	0.4900	1403	1241	1146	333.6
4	Maximum	1.000	0.7900	0.9300	0.9000	2509	2227	2263	537.8
5	Range	0.000	0.3000	0.4600	0.4100	1106	986.4	1116	204.2
6									
7	Mean	1.000	0.6333	0.7533	0.6433	1884	1727	1681	408.9
8	Std. Deviation	0.000	0.1504	0.2479	0.2237	567.2	493.4	559.7	112.2
9	Std. Error of Mean	0.000	0.08686	0.1431	0.1291	327.5	284.8	323.2	64.76

Normality and Lognormality Tests<br>Tabular results	A	B	C	D	E	F	G	H
	NS	CXCL-8	LTB-4	TNF-a	ds-RNA	CXCL-8+ds-RNA	LTB-4+ds-RNA	TNF-a+ds-RNA
	Y	Y	Y	Y	Y	Y	Y	Y
**Test for normal distribution**								
**D'Agostino & Pearson test**								
K2	N too small	N too small	N too small	N too small	N too small	N too small	N too small	N too small
P value								
Passed normality test (alpha=0.05)?								
P value summary								
**Anderson-Darling test**								
A2*	N too small	N too small	N too small	N too small	N too small	N too small	N too small	N too small
P value								
Passed normality test (alpha=0.05)?								
P value summary								
**Shapiro-Wilk test**								
W	Invalid input d	0.9941	0.8611	0.8399	0.9512	0.9994	0.9946	0.8287
P value		0.8533	0.2706	0.2139	0.5747	0.9550	0.8601	0.1851
Passed normality test (alpha=0.05)?		Yes	Yes	Yes	Yes	Yes	Yes	Yes
P value summary		ns	ns	ns	ns	ns	ns	ns
**Kolmogorov-Smirnov test**								
KS distance	N too small	N too small	N too small	N too small	N too small	N too small	N too small	N too small
P value								
Passed normality test (alpha=0.05)?								
P value summary								
**Number of values**	3	3	3	3	3	3	3	3

Ordinary one-way ANOVA<br>ANOVA results					
Table Analyzed	3. Th1 stimulation: PCR TSLP				
Data sets analyzed	A-H				
**ANOVA summary**					
F	21.00				
P value	<0.0001				
P value summary	****				
Significant diff. among means (P < 0.05)?	Yes				
R squared	0.9018				
**Brown-Forsythe test**					
F (DFn, DFd)	2.211 (7, 16)				
P value	0.0895				
P value summary	ns				
Are SDs significantly different (P < 0.05)?	No				
**Bartlett's test**					
Bartlett's statistic (corrected)					
P value					
P value summary					
Are SDs significantly different (P < 0.05)?					
**ANOVA table**	**SS**	**DF**	**MS**	**F (DFn, DFd)**	**P value**
Treatment (between columns)	16373500	7	2339071	F (7, 16) = 21.00	P<0.0001
Residual (within columns)	1782115	16	111382		
Total	18155615	23			
**Data summary**					
Number of treatments (columns)	8				
Number of values (total)	24				

	Ordinary one-way ANOVA<br>Multiple comparisons						
5	**Tukey's multiple comparisons test**	**Mean Diff.**	**95.00% CI of diff.**	**Below threshold?**	**Summary**	**Adjusted P Value**	
6	NS vs. CXCL-8	0.3667	-943.1 to 943.8	No	ns	>0.9999	A-B
7	NS vs. LTB-4	0.2467	-943.2 to 943.7	No	ns	>0.9999	A-C
8	NS vs. TNF-a	0.3567	-943.1 to 943.8	No	ns	>0.9999	A-D
9	NS vs. ds-RNA	-1883	-2826 to -939.2	Yes	****	<0.0001	A-E
10	NS vs. CXCL-8+ds-RNA	-1726	-2670 to -782.8	Yes	***	0.0002	A-F
11	NS vs. LTB-4+ds-RNA	-1680	-2624 to -736.6	Yes	***	0.0003	A-G
12	NS vs. TNF-a+ds-RNA	-407.9	-1351 to 535.5	No	ns	0.7985	A-H
13	CXCL-8 vs. LTB-4	-0.1200	-943.5 to 943.3	No	ns	>0.9999	B-C
14	CXCL-8 vs. TNF-a	-0.01000	-943.4 to 943.4	No	ns	>0.9999	B-D
15	CXCL-8 vs. ds-RNA	-1883	-2826 to -939.6	Yes	****	<0.0001	B-E
16	CXCL-8 vs. CXCL-8+ds-RNA	-1727	-2670 to -783.2	Yes	***	0.0002	B-F
17	CXCL-8 vs. LTB-4+ds-RNA	-1680	-2624 to -737.0	Yes	***	0.0003	B-G
18	CXCL-8 vs. TNF-a+ds-RNA	-408.3	-1352 to 535.1	No	ns	0.7978	B-H
19	LTB-4 vs. TNF-a	0.1100	-943.3 to 943.5	No	ns	>0.9999	C-D
20	LTB-4 vs. ds-RNA	-1883	-2826 to -939.4	Yes	****	<0.0001	C-E
21	LTB-4 vs. CXCL-8+ds-RNA	-1726	-2670 to -783.1	Yes	***	0.0002	C-F
22	LTB-4 vs. LTB-4+ds-RNA	-1680	-2624 to -736.9	Yes	***	0.0003	C-G
23	LTB-4 vs. TNF-a+ds-RNA	-408.2	-1352 to 535.3	No	ns	0.7981	C-H
24	TNF-a vs. ds-RNA	-1883	-2826 to -939.5	Yes	****	<0.0001	D-E
25	TNF-a vs. CXCL-8+ds-RNA	-1727	-2670 to -783.2	Yes	***	0.0002	D-F
26	TNF-a vs. LTB-4+ds-RNA	-1680	-2624 to -737.0	Yes	***	0.0003	D-G
27	TNF-a vs. TNF-a+ds-RNA	-408.3	-1352 to 535.2	No	ns	0.7978	D-H
28	ds-RNA vs. CXCL-8+ds-RNA	156.4	-787.1 to 1100	No	ns	0.9988	E-F
29	ds-RNA vs. LTB-4+ds-RNA	202.5	-740.9 to 1146	No	ns	0.9939	E-G
30	ds-RNA vs. TNF-a+ds-RNA	1475	531.3 to 2418	Yes	**	0.0012	E-H
31	CXCL-8+ds-RNA vs. LTB-4+ds-RNA	46.17	-897.3 to 989.6	No	ns	>0.9999	F-G
32	CXCL-8+ds-RNA vs. TNF-a+ds-RNA	1318	374.9 to 2262	Yes	**	0.0035	F-H
33	LTB-4+ds-RNA vs. TNF-a+ds-RNA	1272	328.7 to 2216	Yes	**	0.0049	G-H

Figure 3 PCR IL-25

NS	CXCL-8	LTB-4	TNF-a	ds-RNA	CXCL-8+ds-RNA	LTB-4+ds-RNA	TNF-a+ds-RNA
1	1,11	0,9	0,9	0,87	0,89	0,85	0,67
1	2,15	3,63	1,62	1,87	2,22	2,94	2,03
1	0,3	0,18	0,34	0,25	0,32	0,35	0,47
1	0,29	0,41	0,49	0,31	0,39	0,4	0,35

	Descriptive statistics	A	B	C	D	E	F	G	H
		NS	CXCL-8	LTB-4	TNF-a	ds-RNA	CXCL-8+ds-RNA	LTB-4+ds-RNA	TNF-a+ds-RNA
		Y	Y	Y	Y	Y	Y	Y	Y
1	Number of values	4	4	4	4	4	4	4	4
2									
3	Minimum	1.000	0.2900	0.1800	0.3400	0.2500	0.3200	0.3500	0.3500
4	Maximum	1.000	2.150	3.630	1.620	1.870	2.220	2.940	2.030
5	Range	0.000	1.860	3.450	1.280	1.620	1.900	2.590	1.680
6									
7	Mean	1.000	0.9625	1.280	0.8375	0.8250	0.9550	1.135	0.8800
8	Std. Deviation	0.000	0.8800	1.595	0.5729	0.7505	0.8807	1.224	0.7779
9	Std. Error of Mean	0.000	0.4400	0.7976	0.2864	0.3753	0.4404	0.6121	0.3890

	Normality and Lognormality Tests<br>Tabular results	A	B	C	D	E	F	G	H
		NS	CXCL-8	LTB-4	TNF-a	ds-RNA	CXCL-8+ds-RNA	LTB-4+ds-RNA	TNF-a+ds-RNA
		Y	Y	Y	Y	Y	Y	Y	Y
1	**Test for normal distribution**								
2	**D'Agostino & Pearson test**								
3	K2	N too small	N too small	N too small	N too small	N too small	N too small	N too small	N too small
4	P value								
5	Passed normality test (alpha=0.05)?								
6	P value summary								
7									
8	**Anderson-Darling test**								
9	A2*	N too small	N too small	N too small	N too small	N too small	N too small	N too small	N too small
10	P value								
11	Passed normality test (alpha=0.05)?								
12	P value summary								
13									
14	**Shapiro-Wilk test**								
15	W	Invalid input d	0.8595	0.7880	0.9127	0.8614	0.8291	0.7653	0.7772
16	P value		0.2584	0.0823	0.4967	0.2653	0.1655	0.0532	0.0672
17	Passed normality test (alpha=0.05)?		Yes	Yes	Yes	Yes	Yes	Yes	Yes
18	P value summary		ns	ns	ns	ns	ns	ns	ns
19									
20	**Kolmogorov-Smirnov test**								
21	KS distance	N too small	N too small	N too small	N too small	N too small	N too small	N too small	N too small
22	P value								
23	Passed normality test (alpha=0.05)?								
24	P value summary								
25									
26	**Number of values**	4	4	4	4	4	4	4	4

ANOVA results × Multiple comparisons ×

	Ordinary one-way ANOVA<br>ANOVA results					
1	Table Analyzed	3. Th1 stimulation: PCR IL-25				
2	Data sets analyzed	A-H				
3						
4	**ANOVA summary**					
5	F	0.1093				
6	P value	0.9971				
7	P value summary	ns				
8	Significant diff. among means (P < 0.05)?	No				
9	R squared	0.03090				
10						
11	**Brown-Forsythe test**					
12	F (DFn, DFd)	0.6051 (7, 24)				
13	P value	0.7459				
14	P value summary	ns				
15	Are SDs significantly different (P < 0.05)?	No				
16						
17	**Bartlett's test**					
18	Bartlett's statistic (corrected)					
19	P value					
20	P value summary					
21	Are SDs significantly different (P < 0.05)?					
22						
23	**ANOVA table**	**SS**	**DF**	**MS**	**F (DFn, DFd)**	**P value**
24	Treatment (between columns)	0.6781	7	0.09688	F (7, 24) = 0.1093	P=0.9971
25	Residual (within columns)	21.27	24	0.8862		
26	Total	21.95	31			

	Ordinary one-way ANOVA<br>Multiple comparisons						
5	**Tukey's multiple comparisons test**	**Mean Diff.**	**95.00% CI of diff.**	**Below threshold?**	**Summary**	**Adjusted P Value**	
6	NS vs. CXCL-8	0.03750	-2.167 to 2.242	No	ns	>0.9999	A-B
7	NS vs. LTB-4	-0.2800	-2.485 to 1.925	No	ns	0.9999	A-C
8	NS vs. TNF-a	0.1625	-2.042 to 2.367	No	ns	>0.9999	A-D
9	NS vs. ds-RNA	0.1750	-2.030 to 2.380	No	ns	>0.9999	A-E
10	NS vs. CXCL-8+ds-RNA	0.04500	-2.160 to 2.250	No	ns	>0.9999	A-F
11	NS vs. LTB-4+ds-RNA	-0.1350	-2.340 to 2.070	No	ns	>0.9999	A-G
12	NS vs. TNF-a+ds-RNA	0.1200	-2.085 to 2.325	No	ns	>0.9999	A-H
13	CXCL-8 vs. LTB-4	-0.3175	-2.522 to 1.887	No	ns	0.9997	B-C
14	CXCL-8 vs. TNF-a	0.1250	-2.080 to 2.330	No	ns	>0.9999	B-D
15	CXCL-8 vs. ds-RNA	0.1375	-2.067 to 2.342	No	ns	>0.9999	B-E
16	CXCL-8 vs. CXCL-8+ds-RNA	0.007500	-2.197 to 2.212	No	ns	>0.9999	B-F
17	CXCL-8 vs. LTB-4+ds-RNA	-0.1725	-2.377 to 2.032	No	ns	>0.9999	B-G
18	CXCL-8 vs. TNF-a+ds-RNA	0.08250	-2.122 to 2.287	No	ns	>0.9999	B-H
19	LTB-4 vs. TNF-a	0.4425	-1.762 to 2.647	No	ns	0.9972	C-D
20	LTB-4 vs. ds-RNA	0.4550	-1.750 to 2.660	No	ns	0.9967	C-E
21	LTB-4 vs. CXCL-8+ds-RNA	0.3250	-1.880 to 2.530	No	ns	0.9996	C-F
22	LTB-4 vs. LTB-4+ds-RNA	0.1450	-2.060 to 2.350	No	ns	>0.9999	C-G
23	LTB-4 vs. TNF-a+ds-RNA	0.4000	-1.805 to 2.605	No	ns	0.9985	C-H
24	TNF-a vs. ds-RNA	0.01250	-2.192 to 2.217	No	ns	>0.9999	D-E
25	TNF-a vs. CXCL-8+ds-RNA	-0.1175	-2.322 to 2.087	No	ns	>0.9999	D-F
26	TNF-a vs. LTB-4+ds-RNA	-0.2975	-2.502 to 1.907	No	ns	0.9998	D-G
27	TNF-a vs. TNF-a+ds-RNA	-0.04250	-2.247 to 2.162	No	ns	>0.9999	D-H
28	ds-RNA vs. CXCL-8+ds-RNA	-0.1300	-2.335 to 2.075	No	ns	>0.9999	E-F
29	ds-RNA vs. LTB-4+ds-RNA	-0.3100	-2.515 to 1.895	No	ns	0.9997	E-G
30	ds-RNA vs. TNF-a+ds-RNA	-0.05500	-2.260 to 2.150	No	ns	>0.9999	E-H
31	CXCL-8+ds-RNA vs. LTB-4+ds-RNA	-0.1800	-2.385 to 2.025	No	ns	>0.9999	F-G
32	CXCL-8+ds-RNA vs. TNF-a+ds-RNA	0.07500	-2.130 to 2.280	No	ns	>0.9999	F-H
33	LTB-4+ds-RNA vs. TNF-a+ds-RNA	0.2550	-1.950 to 2.460	No	ns	>0.9999	G-H

Figure 3 PCR IL-33

NS	CXCL-8	LTB-4	TNF-a	ds-RNA	CXCL-8+ds-RNA	LTB-4+ds-RNA	TNF-a+ds-RNA
1	1,03	1,18	1,39	0,92	0,84	0,84	1,29
1	0,93	1,04	1,03	1,3	1,18	0,84	1,76
1	1,21	0,31	0,89	0,5	0,57	0,5	1,33
1	0,36	0,75	1,29	0,86	0,82	0,57	0,96

	Descriptive statistics	NS	CXCL-8	LTB-4	TNF-a	ds-RNA	CXCL-8+ds-RNA	LTB-4+ds-RNA	TNF-a+ds-RNA
		Y	Y	Y	Y	Y	Y	Y	Y
1	Number of values	4	4	4	4	4	4	4	4
2									
3	Minimum	1.000	0.3600	0.3100	0.8900	0.5000	0.5700	0.5000	0.9600
4	Maximum	1.000	1.210	1.180	1.390	1.300	1.180	0.8400	1.760
5	Range	0.000	0.8500	0.8700	0.5000	0.8000	0.6100	0.3400	0.8000
6									
7	Mean	1.000	0.8825	0.8200	1.150	0.8950	0.8525	0.6875	1.335
8	Std. Deviation	0.000	0.3671	0.3843	0.2304	0.3276	0.2505	0.1784	0.3283
9	Std. Error of Mean	0.000	0.1835	0.1921	0.1152	0.1638	0.1253	0.08920	0.1641

	Normality and Lognormality Tests<br>Tabular results	A	B	C	D	E	F	G	H
		NS	CXCL-8	LTB-4	TNF-a	ds-RNA	CXCL-8+ds-RNA	LTB-4+ds-RNA	TNF-a+ds-RNA
		Y	Y	Y	Y	Y	Y	Y	Y
1	**Test for normal distribution**								
2	**D'Agostino & Pearson test**								
3	K2	N too small	N too small	N too small	N too small	N too small	N too small	N too small	N too small
4	P value								
5	Passed normality test (alpha=0.05)?								
6	P value summary								
7									
8	**Anderson-Darling test**								
9	A2*	N too small	N too small	N too small	N too small	N too small	N too small	N too small	N too small
0	P value								
1	Passed normality test (alpha=0.05)?								
2	P value summary								
3									
4	**Shapiro-Wilk test**								
5	W	Invalid input d	0.8929	0.9425	0.9402	0.9735	0.9484	0.8129	0.9578
6	P value		0.3966	0.6695	0.6554	0.8630	0.7060	0.1273	0.7650
7	Passed normality test (alpha=0.05)?		Yes	Yes	Yes	Yes	Yes	Yes	Yes
8	P value summary		ns	ns	ns	ns	ns	ns	ns
9									
0	**Kolmogorov-Smirnov test**								
1	KS distance	N too small	N too small	N too small	N too small	N too small	N too small	N too small	N too small
2	P value								
3	Passed normality test (alpha=0.05)?								
4	P value summary								
5									
6	**Number of values**	4	4	4	4	4	4	4	4

	Ordinary one-way ANOVA<br>ANOVA results					
1	Table Analyzed	3. Th1 stimulation: PCR IL-33				
2	Data sets analyzed	A-H				
3						
4	**ANOVA summary**					
5	F	2.080				
6	P value	0.0857				
7	P value summary	ns				
8	Significant diff. among means (P < 0.05)?	No				
9	R squared	0.3776				
10						
11	**Brown-Forsythe test**					
12	F (DFn, DFd)	0.9474 (7, 24)				
13	P value	0.4899				
14	P value summary	ns				
15	Are SDs significantly different (P < 0.05)?	No				
16						
17	**Bartlett's test**					
18	Bartlett's statistic (corrected)					
19	P value					
20	P value summary					
21	Are SDs significantly different (P < 0.05)?					
22						
23	**ANOVA table**	**SS**	**DF**	**MS**	**F (DFn, DFd)**	**P value**
24	Treatment (between columns)	1.174	7	0.1677	F (7, 24) = 2.080	P=0.0857
25	Residual (within columns)	1.935	24	0.08064		
26	Total	3.110	31			

ANOVA results | Multiple comparisons

Ordinary one-way ANOVA
Multiple comparisons

5	**Tukey's multiple comparisons test**	**Mean Diff.**	**95.00% CI of diff.**	**Below threshold?**	**Summary**	**Adjusted P Value**	
6	NS vs. CXCL-8	0.1175	-0.5475 to 0.7825	No	ns	0.9988	A-B
7	NS vs. LTB-4	0.1800	-0.4850 to 0.8450	No	ns	0.9836	A-C
8	NS vs. TNF-a	-0.1500	-0.8150 to 0.5150	No	ns	0.9943	A-D
9	NS vs. ds-RNA	0.1050	-0.5600 to 0.7700	No	ns	0.9994	A-E
10	NS vs. CXCL-8+ds-RNA	0.1475	-0.5175 to 0.8125	No	ns	0.9949	A-F
11	NS vs. LTB-4+ds-RNA	0.3125	-0.3525 to 0.9775	No	ns	0.7702	A-G
12	NS vs. TNF-a+ds-RNA	-0.3350	-1.000 to 0.3300	No	ns	0.7062	A-H
13	CXCL-8 vs. LTB-4	0.06250	-0.6025 to 0.7275	No	ns	>0.9999	B-C
14	CXCL-8 vs. TNF-a	-0.2675	-0.9325 to 0.3975	No	ns	0.8775	B-D
15	CXCL-8 vs. ds-RNA	-0.01250	-0.6775 to 0.6525	No	ns	>0.9999	B-E
16	CXCL-8 vs. CXCL-8+ds-RNA	0.03000	-0.6350 to 0.6950	No	ns	>0.9999	B-F
17	CXCL-8 vs. LTB-4+ds-RNA	0.1950	-0.4700 to 0.8600	No	ns	0.9744	B-G
18	CXCL-8 vs. TNF-a+ds-RNA	-0.4525	-1.118 to 0.2125	No	ns	0.3574	B-H
19	LTB-4 vs. TNF-a	-0.3300	-0.9950 to 0.3350	No	ns	0.7208	C-D
20	LTB-4 vs. ds-RNA	-0.07500	-0.7400 to 0.5900	No	ns	>0.9999	C-E
21	LTB-4 vs. CXCL-8+ds-RNA	-0.03250	-0.6975 to 0.6325	No	ns	>0.9999	C-F
22	LTB-4 vs. LTB-4+ds-RNA	0.1325	-0.5325 to 0.7975	No	ns	0.9974	C-G
23	LTB-4 vs. TNF-a+ds-RNA	-0.5150	-1.180 to 0.1500	No	ns	0.2171	C-H
24	TNF-a vs. ds-RNA	0.2550	-0.4100 to 0.9200	No	ns	0.9011	D-E
25	TNF-a vs. CXCL-8+ds-RNA	0.2975	-0.3675 to 0.9625	No	ns	0.8095	D-F
26	TNF-a vs. LTB-4+ds-RNA	0.4625	-0.2025 to 1.128	No	ns	0.3319	D-G
27	TNF-a vs. TNF-a+ds-RNA	-0.1850	-0.8500 to 0.4800	No	ns	0.9808	D-H
28	ds-RNA vs. CXCL-8+ds-RNA	0.04250	-0.6225 to 0.7075	No	ns	>0.9999	E-F
29	ds-RNA vs. LTB-4+ds-RNA	0.2075	-0.4575 to 0.8725	No	ns	0.9643	E-G
30	ds-RNA vs. TNF-a+ds-RNA	-0.4400	-1.105 to 0.2250	No	ns	0.3908	E-H
31	CXCL-8+ds-RNA vs. LTB-4+ds-RNA	0.1650	-0.5000 to 0.8300	No	ns	0.9900	F-G
32	CXCL-8+ds-RNA vs. TNF-a+ds-RNA	-0.4825	-1.148 to 0.1825	No	ns	0.2843	F-H
33	LTB-4+ds-RNA vs. TNF-a+ds-RNA	-0.6475	-1.313 to 0.01754	No	ns	0.0603	G-H

Figure 3 PCR IL-8

NS	CXCL-8	LTB-4	TNF-a	ds-RNA	CXCL-8+ds-RNA	LTB-4+ds-RNA	TNF-a+ds-RNA
1	0,81	0,97	12,96	29,82	34,08	30,04	68,12
1	1,02	1,01	13,43	20,95	20,98	15,476	60,171
1	1,07	0,49	16,92	19,39	11,87	14,24	57,4
1	0,44	0,47	15,43	30,47	34,27	29,86	48,44

	Descriptive statistics	NS	CXCL-8	LTB-4	TNF-a	ds-RNA	CXCL-8+ds-RNA	LTB-4+ds-RNA	TNF-a+ds-RNA
		Y	Y	Y	Y	Y	Y	Y	Y
1	Number of values	4	4	4	4	4	4	4	4
2									
3	Minimum	1.000	0.4400	0.4700	12.96	19.39	11.87	14.24	48.44
4	Maximum	1.000	1.070	1.010	16.92	30.47	34.27	30.04	68.12
5	Range	0.000	0.6300	0.5400	3.960	11.08	22.40	15.80	19.68
6									
7	Mean	1.000	0.8350	0.7350	14.69	25.16	25.30	22.40	58.53
8	Std. Deviation	0.000	0.2864	0.2950	1.835	5.800	10.90	8.728	8.119
9	Std. Error of Mean	0.000	0.1432	0.1475	0.9175	2.900	5.451	4.364	4.059

Normality and Lognormality Tests
Tabular results

		A	B	C	D	E	F	G	H
		NS	CXCL-8	LTB-4	TNF-a	ds-RNA	CXCL-8+ds-RNA	LTB-4+ds-RNA	TNF-a+ds-RNA
		Y	Y	Y	Y	Y	Y	Y	Y
1	**Test for normal distribution**								
2	**D'Agostino & Pearson test**								
3	K2	N too small	N too small	N too small	N too small	N too small	N too small	N too small	N too small
4	P value								
5	Passed normality test (alpha=0.05)?								
6	P value summary								
7									
8	**Anderson-Darling test**								
9	A2*	N too small	N too small	N too small	N too small	N too small	N too small	N too small	N too small
10	P value								
11	Passed normality test (alpha=0.05)?								
12	P value summary								
13									
14	**Shapiro-Wilk test**								
15	W	Invalid input d	0.8896	0.7789	0.9235	0.8187	0.8661	0.7683	0.9892
16	P value		0.3814	0.0694	0.5569	0.1403	0.2827	0.0565	0.9535
17	Passed normality test (alpha=0.05)?		Yes	Yes	Yes	Yes	Yes	Yes	Yes
18	P value summary		ns	ns	ns	ns	ns	ns	ns
19									
20	**Kolmogorov-Smirnov test**								
21	KS distance	N too small	N too small	N too small	N too small	N too small	N too small	N too small	N too small
22	P value								
23	Passed normality test (alpha=0.05)?								
24	P value summary								
25									
26	**Number of values**	4	4	4	4	4	4	4	4

Ordinary one-way ANOVA
ANOVA results

1	Table Analyzed	3. Th1 stimulation: PCR IL-8				
2	Data sets analyzed	A-H				
3						
4	**ANOVA summary**					
5	F	40.74				
6	P value	<0.0001				
7	P value summary	****				
8	Significant diff. among means (P < 0.05)?	Yes				
9	R squared	0.9224				
10						
11	**Brown-Forsythe test**					
12	F (DFn, DFd)	8.912 (7, 24)				
13	P value	<0.0001				
14	P value summary	****				
15	Are SDs significantly different (P < 0.05)?	Yes				
16						
17	**Bartlett's test**					
18	Bartlett's statistic (corrected)					
19	P value					
20	P value summary					
21	Are SDs significantly different (P < 0.05)?					
22						
23	**ANOVA table**	**SS**	**DF**	**MS**	**F (DFn, DFd)**	**P value**
24	Treatment (between columns)	10627	7	1518	F (7, 24) = 40.74	P<0.0001
25	Residual (within columns)	894.4	24	37.27		
26	Total	11522	31			
27						
28	**Data summary**					
29	Number of treatments (columns)	8				
30	Number of values (total)	32				

	Ordinary one-way ANOVA<br>Multiple comparisons						
5	**Tukey's multiple comparisons test**	**Mean Diff.**	**95.00% CI of diff.**	**Below threshold?**	**Summary**	**Adjusted P Value**	
6	NS vs. CXCL-8	0.1650	-14.13 to 14.46	No	ns	>0.9999	A-B
7	NS vs. LTB-4	0.2650	-14.03 to 14.56	No	ns	>0.9999	A-C
8	NS vs. TNF-a	-13.69	-27.98 to 0.6114	No	ns	0.0676	A-D
9	NS vs. ds-RNA	-24.16	-38.45 to -9.861	Yes	***	0.0002	A-E
10	NS vs. CXCL-8+ds-RNA	-24.30	-38.60 to -10.00	Yes	***	0.0002	A-F
11	NS vs. LTB-4+ds-RNA	-21.40	-35.70 to -7.108	Yes	**	0.0010	A-G
12	NS vs. TNF-a+ds-RNA	-57.53	-71.83 to -43.24	Yes	****	<0.0001	A-H
13	CXCL-8 vs. LTB-4	0.1000	-14.20 to 14.40	No	ns	>0.9999	B-C
14	CXCL-8 vs. TNF-a	-13.85	-28.15 to 0.4464	No	ns	0.0624	B-D
15	CXCL-8 vs. ds-RNA	-24.32	-38.62 to -10.03	Yes	***	0.0002	B-E
16	CXCL-8 vs. CXCL-8+ds-RNA	-24.47	-38.76 to -10.17	Yes	***	0.0002	B-F
17	CXCL-8 vs. LTB-4+ds-RNA	-21.57	-35.87 to -7.273	Yes	***	0.0009	B-G
18	CXCL-8 vs. TNF-a+ds-RNA	-57.70	-71.99 to -43.40	Yes	****	<0.0001	B-H
19	LTB-4 vs. TNF-a	-13.95	-28.25 to 0.3464	No	ns	0.0594	C-D
20	LTB-4 vs. ds-RNA	-24.42	-38.72 to -10.13	Yes	***	0.0002	C-E
21	LTB-4 vs. CXCL-8+ds-RNA	-24.57	-38.86 to -10.27	Yes	***	0.0002	C-F
22	LTB-4 vs. LTB-4+ds-RNA	-21.67	-35.97 to -7.373	Yes	***	0.0009	C-G
23	LTB-4 vs. TNF-a+ds-RNA	-57.80	-72.09 to -43.50	Yes	****	<0.0001	C-H
24	TNF-a vs. ds-RNA	-10.47	-24.77 to 3.824	No	ns	0.2739	D-E
25	TNF-a vs. CXCL-8+ds-RNA	-10.62	-24.91 to 3.681	No	ns	0.2595	D-F
26	TNF-a vs. LTB-4+ds-RNA	-7.719	-22.02 to 6.577	No	ns	0.6333	D-G
27	TNF-a vs. TNF-a+ds-RNA	-43.85	-58.14 to -29.55	Yes	****	<0.0001	D-H
28	ds-RNA vs. CXCL-8+ds-RNA	-0.1425	-14.44 to 14.15	No	ns	>0.9999	E-F
29	ds-RNA vs. LTB-4+ds-RNA	2.754	-11.54 to 17.05	No	ns	0.9979	E-G
30	ds-RNA vs. TNF-a+ds-RNA	-33.38	-47.67 to -19.08	Yes	****	<0.0001	E-H
31	CXCL-8+ds-RNA vs. LTB-4+ds-RNA	2.896	-11.40 to 17.19	No	ns	0.9971	F-G
32	CXCL-8+ds-RNA vs. TNF-a+ds-RNA	-33.23	-47.53 to -18.94	Yes	****	<0.0001	F-H
33	LTB-4+ds-RNA vs. TNF-a+ds-RNA	-36.13	-50.43 to -21.83	Yes	****	<0.0001	G-H

Figure 3 WB IL-8

control	ds-RNA	TNF-a+ds-RNA
1	1,18291158	1,265084364
1	1,15109134	1,363692233
1	1,14261993	1,440286603

	Descriptive statistics	control	ds-RNA	TNF-a+ds-RNA
		Y	Y	Y
1	Number of values	3	3	3
2				
3	Minimum	1.000	1.143	1.265
4	Maximum	1.000	1.183	1.440
5	Range	0.000	0.04029	0.1752
6				
7	Mean	1.000	1.159	1.356
8	Std. Deviation	0.000	0.02124	0.08783
9	Std. Error of Mean	0.000	0.01226	0.05071

	Normality and Lognormality Tests<br>Tabular results	A<br>control	B<br>ds-RNA	C<br>TNF-a+ds-RNA
		Y	Y	Y
1	**Test for normal distribution**			
2	**D'Agostino & Pearson test**			
3	K2	N too small	N too small	N too small
4	P value			
5	Passed normality test (alpha=0.05)?			
6	P value summary			
7				
8	**Anderson-Darling test**			
9	A2*	N too small	N too small	N too small
10	P value			
11	Passed normality test (alpha=0.05)?			
12	P value summary			
13				
14	**Shapiro-Wilk test**			
15	W	Invalid input d	0.8993	0.9948
16	P value		0.3834	0.8617
17	Passed normality test (alpha=0.05)?		Yes	Yes
18	P value summary		ns	ns
19				
20	**Kolmogorov-Smirnov test**			
21	KS distance	N too small	N too small	N too small
22	P value			
23	Passed normality test (alpha=0.05)?			
24	P value summary			
25				
26	**Number of values**	3	3	3

	Ordinary one-way ANOVA<br>ANOVA results					
1	Table Analyzed	3. Th1 stimulation: Protein IL-8 3 groups				
2	Data sets analyzed	A-C				
3						
4	**ANOVA summary**					
5	F	35.13				
6	P value	0.0005				
7	P value summary	***				
8	Significant diff. among means (P < 0.05)?	Yes				
9	R squared	0.9213				
0						
1	**Brown-Forsythe test**					
2	F (DFn, DFd)	2.854 (2, 6)				
3	P value	0.1346				
4	P value summary	ns				
5	Are SDs significantly different (P < 0.05)?	No				
6						
7	**Bartlett's test**					
8	Bartlett's statistic (corrected)					
9	P value					
0	P value summary					
1	Are SDs significantly different (P < 0.05)?					
2						
3	**ANOVA table**	**SS**	**DF**	**MS**	**F (DFn, DFd)**	**P value**
4	Treatment (between columns)	0.1912	2	0.09561	F (2, 6) = 35.13	P=0.0005
5	Residual (within columns)	0.01633	6	0.002722		
6	Total	0.2076	8			
7						
8	**Data summary**					
9	Number of treatments (columns)	3				
0	Number of values (total)	9				

Ordinary one-way ANOVA<br>Multiple comparisons								
Number of families	1							
Number of comparisons per family	3							
Alpha	0.05							
**Tukey's multiple comparisons test**	**Mean Diff.**	**95.00% CI of diff.**	**Below threshold?**	**Summary**	**Adjusted P Value**			
control vs. ds-RNA	-0.1589	-0.2896 to -0.02817	Yes	*	0.0227	A-B		
control vs. TNF-a+ds-RNA	-0.3564	-0.4871 to -0.2257	Yes	***	0.0004	A-C		
ds-RNA vs. TNF-a+ds-RNA	-0.1975	-0.3282 to -0.06678	Yes	**	0.0085	B-C		
**Test details**	**Mean 1**	**Mean 2**	**Mean Diff.**	**SE of diff.**	**n1**	**n2**	**q**	**DF**
control vs. ds-RNA	1.000	1.159	-0.1589	0.04260	3	3	5.274	6
control vs. TNF-a+ds-RNA	1.000	1.356	-0.3564	0.04260	3	3	11.83	6
ds-RNA vs. TNF-a+ds-RNA	1.159	1.356	-0.1975	0.04260	3	3	6.556	6
**Compact letter display**								
TNF-a+ds-RNA	A							
ds-RNA	B							
control	C							

Figure 3 WB TSLP

control	ds-RNA	TNF-a+ds-RNA
1	1,32947896	1,162047389
1	1,35586858	1,070152746
1	1,4894755	1,177804034

Descriptive statistics	control	ds-RNA	TNF-a+ds-RNA
	Y	Y	Y
Number of values	3	3	3
Minimum	1.000	1.329	1.070
Maximum	1.000	1.489	1.178
Range	0.000	0.1600	0.1077
Mean	1.000	1.392	1.137
Std. Deviation	0.000	0.08578	0.05814
Std. Error of Mean	0.000	0.04952	0.03357

	Normality and Lognormality Tests<br>Tabular results	A<br>control	B<br>ds-RNA	C<br>TNF-a+ds-RN
		Y	Y	Y
1	**Test for normal distribution**			
2	**D'Agostino & Pearson test**			
3	K2	N too small	N too small	N too small
4	P value			
5	Passed normality test (alpha=0.05)?			
6	P value summary			
7				
8	**Anderson-Darling test**			
9	A2*	N too small	N too small	N too small
10	P value			
11	Passed normality test (alpha=0.05)?			
12	P value summary			
13				
14	**Shapiro-Wilk test**			
15	W	Invalid input d	0.8698	0.8571
16	P value		0.2950	0.2596
17	Passed normality test (alpha=0.05)?		Yes	Yes
18	P value summary		ns	ns
19				
20	**Kolmogorov-Smirnov test**			
21	KS distance	N too small	N too small	N too small
22	P value			
23	Passed normality test (alpha=0.05)?			
24	P value summary			
25				
26	**Number of values**	3	3	3

	Ordinary one-way ANOVA<br>ANOVA results					
1	Table Analyzed	3. Th1 stimulation: Protein TSLP 3 groups				
2	Data sets analyzed	A-C				
3						
4	**ANOVA summary**					
5	F	33.11				
6	P value	0.0006				
7	P value summary	***				
8	Significant diff. among means (P < 0.05)?	Yes				
9	R squared	0.9169				
10						
11	**Brown-Forsythe test**					
12	F (DFn, DFd)	0.8966 (2, 6)				
13	P value	0.4564				
14	P value summary	ns				
15	Are SDs significantly different (P < 0.05)?	No				
16						
17	**Bartlett's test**					
18	Bartlett's statistic (corrected)					
19	P value					
20	P value summary					
21	Are SDs significantly different (P < 0.05)?					
22						
23	**ANOVA table**	**SS**	**DF**	**MS**	**F (DFn, DFd)**	**P value**
24	Treatment (between columns)	0.2370	2	0.1185	F (2, 6) = 33.11	P=0.0006
25	Residual (within columns)	0.02148	6	0.003579		
26	Total	0.2585	8			
27						
28	**Data summary**					
29	Number of treatments (columns)	3				
30	Number of values (total)	9				

	Ordinary one-way ANOVA<br>Multiple comparisons							
1	Number of families	1						
2	Number of comparisons per family	3						
3	Alpha	0.05						
4								
5	**Tukey's multiple comparisons test**	**Mean Diff.**	**95.00% CI of diff.**	**Below threshold?**	**Summary**	**Adjusted P Value**		
6	control vs. ds-RNA	-0.3916	-0.5415 to -0.2417	Yes	***	0.0005	A-B	
7	control vs. TNF-a+ds-RNA	-0.1367	-0.2865 to 0.01321	No	ns	0.0700	A-C	
8	ds-RNA vs. TNF-a+ds-RNA	0.2549	0.1051 to 0.4048	Yes	**	0.0048	B-C	
9								
10	**Test details**	**Mean 1**	**Mean 2**	**Mean Diff.**	**SE of diff.**	**n1**	**n2**	**q**
11	control vs. ds-RNA	1.000	1.392	-0.3916	0.04885	3	3	11.34
12	control vs. TNF-a+ds-RNA	1.000	1.137	-0.1367	0.04885	3	3	3.957
13	ds-RNA vs. TNF-a+ds-RNA	1.392	1.137	0.2549	0.04885	3	3	7.381
14								
15	**Compact letter display**							
16	ds-RNA	A						
17	TNF-a+ds-RNA	B						
18	control	B						

Figure 4 DUPILUMAB+IL-4 PCR IL-8

NS	ds-RNA	IL-4+ds-RNA	dup 10+IL-4+ds-RNA	dup 100+IL-4+ds-RNA	dup 1000+IL-4+ds-RNA
1	13,27	35,41	26,43	22,77	20,46
1	18,04	33,64	31,15	26,52	21,78
1	21,87	31,69	36,71	34,97	27,63

	Descriptive statistics	A<br>NS<br>Y	B<br>ds-RNA<br>Y	C<br>IL-4+ds-RNA<br>Y	D<br>dup 10+IL-4+ds-RNA<br>Y	E<br>dup 100+IL-4+ds-RNA<br>Y	F<br>dup 1000+IL-4+ds-RNA<br>Y
1	Number of values	3	3	3	3	3	3
2							
3	Minimum	1.000	13.27	31.69	26.43	22.77	20.46
4	Maximum	1.000	21.87	35.41	36.71	34.97	27.63
5	Range	0.000	8.600	3.720	10.28	12.20	7.170
6							
7	Mean	1.000	17.73	33.58	31.43	28.09	23.29
8	Std. Deviation	0.000	4.309	1.861	5.146	6.249	3.816
9	Std. Error of Mean	0.000	2.488	1.074	2.971	3.608	2.203

	Normality and Lognormality Tests<br>Tabular results	A	B	C	D	E	F
		NS	ds-RNA	IL-4+ds-RNA	dup 10+IL-4+ds-RNA	dup 100+IL-4+ds-RNA	dup 1000+IL-4+ds-RNA
		Y	Y	Y	Y	Y	Y
1	**Test for normal distribution**						
2	**D'Agostino & Pearson test**						
3	K2	N too small	N too small	N too small	N too small	N too small	N too small
4	P value						
5	Passed normality test (alpha=0.05)?						
6	P value summary						
7							
8	**Anderson-Darling test**						
9	A2*	N too small	N too small	N too small	N too small	N too small	N too small
10	P value						
11	Passed normality test (alpha=0.05)?						
12	P value summary						
13							
14	**Shapiro-Wilk test**						
15	W	Invalid input d	0.9960	0.9992	0.9978	0.9529	0.8826
16	P value		0.8796	0.9467	0.9100	0.5820	0.3320
17	Passed normality test (alpha=0.05)?		Yes	Yes	Yes	Yes	Yes
18	P value summary		ns	ns	ns	ns	ns
19							
20	**Kolmogorov-Smirnov test**						
21	KS distance	N too small	N too small	N too small	N too small	N too small	N too small
22	P value						
23	Passed normality test (alpha=0.05)?						
24	P value summary						
25							
26	**Number of values**	3	3	3	3	3	3

	Ordinary one-way ANOVA<br>ANOVA results					
1	Table Analyzed	4. Dupilumab and IL-4: PCR IL-8 3 data				
2	Data sets analyzed	A-F				
3						
4	**ANOVA summary**					
5	F	25.36				
6	P value	<0.0001				
7	P value summary	****				
8	Significant diff. among means (P < 0.05)?	Yes				
9	R squared	0.9135				
10						
11	**Brown-Forsythe test**					
12	F (DFn, DFd)	0.9131 (5, 12)				
13	P value	0.5045				
14	P value summary	ns				
15	Are SDs significantly different (P < 0.05)?	No				
16						
17	**Bartlett's test**					
18	Bartlett's statistic (corrected)					
19	P value					
20	P value summary					
21	Are SDs significantly different (P < 0.05)?					
22						
23	**ANOVA table**	**SS**	**DF**	**MS**	**F (DFn, DFd)**	**P value**
24	Treatment (between columns)	2158	5	431.6	F (5, 12) = 25.36	P<0.0001
25	Residual (within columns)	204.2	12	17.02		
26	Total	2362	17			
27						
28	**Data summary**					
29	Number of treatments (columns)	6				
30	Number of values (total)	18				

ANOVA results | Multiple comparisons

Ordinary one-way ANOVA Multiple comparisons						
2 Number of comparisons per family	15					
3 Alpha	0.05					
4						
5 **Tukey's multiple comparisons test**	**Mean Diff.**	**95.00% CI of diff.**	**Below threshold?**	**Summary**	**Adjusted P Value**	
6 NS vs. ds-RNA	-16.73	-28.04 to -5.412	Yes	**	0.0034	A-B
7 NS vs. IL-4+ds-RNA	-32.58	-43.89 to -21.27	Yes	****	<0.0001	A-C
8 NS vs. dup 10+IL-4+ds-RNA	-30.43	-41.74 to -19.12	Yes	****	<0.0001	A-D
9 NS vs. dup 100+IL-4+ds-RNA	-27.09	-38.40 to -15.77	Yes	****	<0.0001	A-E
10 NS vs. dup 1000+IL-4+ds-RNA	-22.29	-33.60 to -10.98	Yes	***	0.0003	A-F
11 ds-RNA vs. IL-4+ds-RNA	-15.85	-27.17 to -4.539	Yes	**	0.0052	B-C
12 ds-RNA vs. dup 10+IL-4+ds-RNA	-13.70	-25.02 to -2.389	Yes	*	0.0151	B-D
13 ds-RNA vs. dup 100+IL-4+ds-RNA	-10.36	-21.67 to 0.9543	No	ns	0.0800	B-E
14 ds-RNA vs. dup 1000+IL-4+ds-RNA	-5.563	-16.88 to 5.751	No	ns	0.5838	B-F
15 IL-4+ds-RNA vs. dup 10+IL-4+ds-RNA	2.150	-9.164 to 13.46	No	ns	0.9855	C-D
16 IL-4+ds-RNA vs. dup 100+IL-4+ds-RNA	5.493	-5.821 to 16.81	No	ns	0.5957	C-E
17 IL-4+ds-RNA vs. dup 1000+IL-4+ds-RNA	10.29	-1.024 to 21.60	No	ns	0.0828	C-F
18 dup 10+IL-4+ds-RNA vs. dup 100+IL-4+ds-RNA	3.343	-7.971 to 14.66	No	ns	0.9117	D-E
19 dup 10+IL-4+ds-RNA vs. dup 1000+IL-4+ds-RNA	8.140	-3.174 to 19.45	No	ns	0.2245	D-F
20 dup 100+IL-4+ds-RNA vs. dup 1000+IL-4+ds-RNA	4.797	-6.518 to 16.11	No	ns	0.7135	E-F

Figure 4 DUPILUMAB+IL-4 PCR TSLP

NS	ds-RNA	IL-4+ds-RNA	dup 10+IL-4+ds-RNA	dup 100+IL-4+ds-RNA	dup 1000+IL-4+ds-RNA
1	1699,62	2370,54	1868,93	1799,03	2324,98
1	1624,75	1895,02	2036,67	1858,6	1361,52
1	1295,24	1414,43	1430,2	1441,15	1319,71

Descriptive statistics	NS	ds-RNA	IL-4+ds-RNA	dup 10+IL-4+ds-RNA	dup 100+IL-4+ds-RNA	dup 1000+IL-4+ds-RNA
	Y	Y	Y	Y	Y	Y
1 Number of values	3	3	3	3	3	3
2						
3 Minimum	1.000	1295	1414	1430	1441	1320
4 Maximum	1.000	1700	2371	2037	1859	2325
5 Range	0.000	404.4	956.1	606.5	417.5	1005
6						
7 Mean	1.000	1540	1893	1779	1700	1669
8 Std. Deviation	0.000	215.1	478.1	313.2	225.8	568.7
9 Std. Error of Mean	0.000	124.2	276.0	180.8	130.4	328.3

Normality and Lognormality Tests<br>Tabular results	A	B	C	D	E	F
	NS	ds-RNA	IL-4+ds-RNA	dup 10+IL-4+ds-RNA	dup 100+IL-4+ds-RNA	dup 1000+IL-4+ds-RNA
	Y	Y	Y	Y	Y	Y
**Test for normal distribution**						
**D'Agostino & Pearson test**						
K2	N too small	N too small	N too small	N too small	N too small	N too small
P value						
Passed normality test (alpha=0.05)?						
P value summary						
**Anderson-Darling test**						
A2*	N too small	N too small	N too small	N too small	N too small	N too small
P value						
Passed normality test (alpha=0.05)?						
P value summary						
**Shapiro-Wilk test**						
W	Invalid input d	0.8833	1.000	0.9376	0.8545	0.7811
P value		0.3340	0.9942	0.5178	0.2527	0.0702
Passed normality test (alpha=0.05)?		Yes	Yes	Yes	Yes	Yes
P value summary		ns	ns	ns	ns	ns
**Kolmogorov-Smirnov test**						
KS distance	N too small	N too small	N too small	N too small	N too small	N too small
P value						
Passed normality test (alpha=0.05)?						
P value summary						
**Number of values**	3	3	3	3	3	3

Ordinary one-way ANOVA<br>ANOVA results					
Table Analyzed	4. Dupilumab and IL-4: PCR TSLP 3 da				
Data sets analyzed	A-F				
Distribution assumption	Normal (Gaussian)				
**ANOVA summary**					
F	12.14				
P value	0.0002				
P value summary	***				
Significant diff. among means (P < 0.05)?	Yes				
R squared	0.8349				
**Brown-Forsythe test**					
F (DFn, DFd)	0.5859 (5, 12)				
P value	0.7110				
P value summary	ns				
Are SDs significantly different (P < 0.05)?	No				
**Bartlett's test**					
Bartlett's statistic (corrected)					
P value					
P value summary					
Are SDs significantly different (P < 0.05)?					
**ANOVA table**	**SS**	**DF**	**MS**	**F (DFn, DFd)**	**P value**
Treatment (between columns)	7559954	5	1511991	F (5, 12) = 12.14	P=0.0002
Residual (within columns)	1494609	12	124551		
Total	9054563	17			
**Data summary**					
Number of treatments (columns)	6				
Number of values (total)	18				

ANVOVA results | Multiple comparisons

Ordinary one-way ANOVA<br>Multiple comparisons							
1	Number of families	1					
2	Number of comparisons per family	15					
3	Alpha	0.05					
4							
5	**Tukey's multiple comparisons test**	**Mean diff.**	**95.00% CI of diff.**	**Below threshold?**	**Summary**	**Adjusted P Value**	
6	NS vs. ds-RNA	-1539	-2507 to -571.0	Yes	**	0.0019	A-B
7	NS vs. IL-4+ds-RNA	-1892	-2860 to -924.4	Yes	***	0.0003	A-C
8	NS vs. dup 10+IL-4+ds-RNA	-1778	-2745 to -809.7	Yes	***	0.0005	A-D
9	NS vs. dup 100+IL-4+ds-RNA	-1699	-2666 to -730.7	Yes	***	0.0008	A-E
10	NS vs. dup 1000+IL-4+ds-RNA	-1668	-2636 to -699.8	Yes	***	0.0009	A-F
11	ds-RNA vs. IL-4+ds-RNA	-353.5	-1321 to 614.4	No	ns	0.8163	B-C
12	ds-RNA vs. dup 10+IL-4+ds-RNA	-238.7	-1207 to 729.2	No	ns	0.9563	B-D
13	ds-RNA vs. dup 100+IL-4+ds-RNA	-159.7	-1128 to 808.2	No	ns	0.9923	B-E
14	ds-RNA vs. dup 1000+IL-4+ds-RNA	-128.9	-1097 to 839.0	No	ns	0.9972	B-F
15	IL-4+ds-RNA vs. dup 10+IL-4+ds-RNA	114.7	-853.2 to 1083	No	ns	0.9984	C-D
16	IL-4+ds-RNA vs. dup 100+IL-4+ds-RNA	193.7	-774.2 to 1162	No	ns	0.9818	C-E
17	IL-4+ds-RNA vs. dup 1000+IL-4+ds-RNA	224.6	-743.3 to 1192	No	ns	0.9660	C-F
18	dup 10+IL-4+ds-RNA vs. dup 100+IL-4+ds-RNA	79.01	-888.9 to 1047	No	ns	0.9997	D-E
19	dup 10+IL-4+ds-RNA vs. dup 1000+IL-4+ds-RNA	109.9	-858.0 to 1078	No	ns	0.9987	D-F
20	dup 100+IL-4+ds-RNA vs. dup 1000+IL-4+ds-RNA	30.86	-937.0 to 998.7	No	ns	>0.9999	E-F

Figure 4 DUPILUMAB+IL4 WB IL-8

control	ds-RNA	IL-4+ds-RNA	dup 1000+IL-4+ds-RNA
1	1,209544946	1,309421926	0,823794087
1	1,119993559	1,311759966	0,91822429
1	1,077318308	1,242013082	0,834037378

Descriptive statistics		control	ds-RNA	IL-4+ds-RNA	dup 1000+IL-4+ds-RNA
		Y	Y	Y	Y
1	Number of values	3	3	3	3
2					
3	Minimum	1.000	1.077	1.242	0.8238
4	Maximum	1.000	1.210	1.312	0.9182
5	Range	0.000	0.1322	0.06975	0.09443
6					
7	Mean	1.000	1.136	1.288	0.8587
8	Std. Deviation	0.000	0.06748	0.03961	0.05182
9	Std. Error of Mean	0.000	0.03896	0.02287	0.02992

	Normality and Lognormality Tests<br>Tabular results	A<br>control	B<br>ds-RNA	C<br>IL-4+ds-RNA	D<br>dup 1000+IL-4+ds-RNA
		Y	Y	Y	Y
1	**Test for normal distribution**				
2	**D'Agostino & Pearson test**				
3	K2	N too small	N too small	N too small	N too small
4	P value				
5	Passed normality test (alpha=0.05)?				
6	P value summary				
7					
8	**Anderson-Darling test**				
9	A2*	N too small	N too small	N too small	N too small
10	P value				
11	Passed normality test (alpha=0.05)?				
12	P value summary				
13					
14	**Shapiro-Wilk test**				
15	W	Invalid input d	0.9598	0.7751	0.8303
16	P value		0.6144	0.0564	0.1891
17	Passed normality test (alpha=0.05)?		Yes	Yes	Yes
18	P value summary		ns	ns	ns
19					
20	**Kolmogorov-Smirnov test**				
21	KS distance	N too small	N too small	N too small	N too small
22	P value				
23	Passed normality test (alpha=0.05)?				
24	P value summary				
25					
26	**Number of values**	3	3	3	3

	Ordinary one-way ANOVA<br>ANOVA results					
1	Table Analyzed	4. Dupilumab and IL4: Protein IL-8 (PANEL 2)				
2	Data sets analyzed	A-D				
3						
4	**ANOVA summary**					
5	F	45.99				
6	P value	<0.0001				
7	P value summary	****				
8	Significant diff. among means (P < 0.05)?	Yes				
9	R squared	0.9452				
10						
11	**Brown-Forsythe test**					
12	F (DFn, DFd)	0.7408 (3, 8)				
13	P value	0.5569				
14	P value summary	ns				
15	Are SDs significantly different (P < 0.05)?	No				
16						
17	**Bartlett's test**					
18	Bartlett's statistic (corrected)					
19	P value					
20	P value summary					
21	Are SDs significantly different (P < 0.05)?					
22						
23	**ANOVA table**	**SS**	**DF**	**MS**	**F (DFn, DFd)**	**P value**
24	Treatment (between columns)	0.3038	3	0.1013	F (3, 8) = 45.99	P<0.0001
25	Residual (within columns)	0.01762	8	0.002202		
26	Total	0.3214	11			
27						
28	**Data summary**					
29	Number of treatments (columns)	4				
30	Number of values (total)	12				

Ordinary one-way ANOVA<br>Multiple comparisons								
Number of families	1							
Number of comparisons per family	6							
Alpha	0.05							
**Tukey's multiple comparisons test**	**Mean Diff.**	**95.00% CI of diff.**	**Below threshold?**	**Summary**	**Adjusted P Value**			
control vs. ds-RNA	-0.1356	-0.2583 to -0.01292	Yes	*	0.0312	A-B		
control vs. IL-4+ds-RNA	-0.2877	-0.4104 to -0.1650	Yes	***	0.0003	A-C		
control vs. dup 1000+IL-4+ds-RNA	0.1413	0.01862 to 0.2640	Yes	*	0.0254	A-D		
ds-RNA vs. IL-4+ds-RNA	-0.1521	-0.2748 to -0.02942	Yes	*	0.0173	B-C		
ds-RNA vs. dup 1000+IL-4+ds-RNA	0.2769	0.1542 to 0.3996	Yes	***	0.0004	B-D		
IL-4+ds-RNA vs. dup 1000+IL-4+ds-RNA	0.4290	0.3063 to 0.5517	Yes	****	<0.0001	C-D		
**Test details**	**Mean 1**	**Mean 2**	**Mean Diff.**	**SE of diff.**	**n1**	**n2**	**q**	**DF**
control vs. ds-RNA	1.000	1.136	-0.1356	0.03831	3	3	5.006	8
control vs. IL-4+ds-RNA	1.000	1.288	-0.2877	0.03831	3	3	10.62	8
control vs. dup 1000+IL-4+ds-RNA	1.000	0.8587	0.1413	0.03831	3	3	5.216	8
ds-RNA vs. IL-4+ds-RNA	1.136	1.288	-0.1521	0.03831	3	3	5.615	8
ds-RNA vs. dup 1000+IL-4+ds-RNA	1.136	0.8587	0.2769	0.03831	3	3	10.22	8
IL-4+ds-RNA vs. dup 1000+IL-4+ds-RNA	1.288	0.8587	0.4290	0.03831	3	3	15.84	8

Figure 4 DUPILUMAB+IL4 WB TSLP

control	ds-RNA	IL-4+ds-RNA	dup 1000+IL-4+ds-RNA
1	1,264420955	1,586595597	1,38408201
1	1,219846342	1,642560813	1,150456836
1	1,481148889	1,807792312	1,135089131
1	1,18294498	1,716581006	1,532366618

Descriptive statistics	control	ds-RNA	IL-4+ds-RNA	dup 1000+IL-4+ds-RNA
	Y	Y	Y	Y
Number of values	4	4	4	4
Minimum	1.000	1.183	1.587	1.135
Maximum	1.000	1.481	1.808	1.532
Range	0.000	0.2982	0.2212	0.3973
Mean	1.000	1.287	1.688	1.300
Std. Deviation	0.000	0.1336	0.09577	0.1920
Std. Error of Mean	0.000	0.06680	0.04788	0.09601

	Normality and Lognormality Tests<br>Tabular results	A<br>control	B<br>ds-RNA	C<br>IL-4+ds-RNA	D<br>dup 1000+IL-4+ds-RNA
		Y	Y	Y	Y
1	**Test for normal distribution**				
2	**D'Agostino & Pearson test**				
3	K2	N too small	N too small	N too small	N too small
4	P value				
5	Passed normality test (alpha=0.05)?				
6	P value summary				
7					
8	**Anderson-Darling test**				
9	A2*	N too small	N too small	N too small	N too small
10	P value				
11	Passed normality test (alpha=0.05)?				
12	P value summary				
13					
14	**Shapiro-Wilk test**				
15	W	Invalid input d	0.8423	0.9815	0.8794
16	P value		0.2021	0.9108	0.3360
17	Passed normality test (alpha=0.05)?		Yes	Yes	Yes
18	P value summary		ns	ns	ns
19					
20	**Kolmogorov-Smirnov test**				
21	KS distance	N too small	N too small	N too small	N too small
22	P value				
23	Passed normality test (alpha=0.05)?				
24	P value summary				
25					
26	**Number of values**	4	4	4	4

	Ordinary one-way ANOVA<br>ANOVA results					
1	Table Analyzed	4. Dupilumab and IL4: Protein TSLP (PANEL 2)				
2	Data sets analyzed	A-D				
3						
4	**ANOVA summary**					
5	F	20.00				
6	P value	<0.0001				
7	P value summary	****				
8	Significant diff. among means (P < 0.05)?	Yes				
9	R squared	0.8333				
10						
11	**Brown-Forsythe test**					
12	F (DFn, DFd)	3.719 (3, 12)				
13	P value	0.0423				
14	P value summary	*				
15	Are SDs significantly different (P < 0.05)?	Yes				
16						
17	**Bartlett's test**					
18	Bartlett's statistic (corrected)					
19	P value					
20	P value summary					
21	Are SDs significantly different (P < 0.05)?					
22						
23	**ANOVA table**	**SS**	**DF**	**MS**	**F (DFn, DFd)**	**P value**
24	Treatment (between columns)	0.9583	3	0.3194	F (3, 12) = 20.00	P<0.0001
25	Residual (within columns)	0.1917	12	0.01597		
26	Total	1.150	15			
27						
28	**Data summary**					
29	Number of treatments (columns)	4				
30	Number of values (total)	16				

	Descriptive statistics	A<br>NS	B<br>ds-RNA	C<br>IL-13+ds-RNA	D<br>dup 10+IL-13+ds-RNA	E<br>dup 100+IL-13+ds-RNA	F<br>dup 1000+IL-13+ds-RNA
		Y	Y	Y	Y	Y	Y
1	Number of values	4	4	4	4	4	4
2							
3	Minimum	1.000	3795	13262	8046	4618	5931
4	Maximum	1.000	24937	82323	33065	22947	26616
5	Range	0.000	21142	69061	25019	18329	20685
6							
7	Mean	1.000	14345	40519	21205	13222	14387
8	Std. Deviation	0.000	12102	33001	12043	9512	10227
9	Std. Error of Mean	0.000	6051	16500	6021	4756	5113

ANOVA results | Multiple comparisons

Ordinary one-way ANOVA<br>Multiple comparisons									
1	Number of families	1							
2	Number of comparisons per family	6							
3	Alpha	0.05							
4									
5	**Tukey's multiple comparisons test**	**Mean Diff.**	**95.00% CI of diff.**	**Below threshold?**	**Summary**	**Adjusted P Value**			
6	control vs. ds-RNA	-0.2871	-0.5524 to -0.02177	Yes	*	0.0327	A-B		
7	control vs. IL-4+ds-RNA	-0.6884	-0.9537 to -0.4231	Yes	****	<0.0001	A-C		
8	control vs. dup 1000+IL-4+ds-RNA	-0.3005	-0.5658 to -0.03518	Yes	*	0.0252	A-D		
9	ds-RNA vs. IL-4+ds-RNA	-0.4013	-0.6666 to -0.1360	Yes	**	0.0036	B-C		
10	ds-RNA vs. dup 1000+IL-4+ds-RNA	-0.01341	-0.2787 to 0.2519	No	ns	0.9987	B-D		
11	IL-4+ds-RNA vs. dup 1000+IL-4+ds-RNA	0.3879	0.1226 to 0.6532	Yes	**	0.0046	C-D		
12									
13	**Test details**	**Mean 1**	**Mean 2**	**Mean Diff.**	**SE of diff.**	**n1**	**n2**	**q**	**DF**
14	control vs. ds-RNA	1.000	1.287	-0.2871	0.08937	4	4	4.543	12
15	control vs. IL-4+ds-RNA	1.000	1.688	-0.6884	0.08937	4	4	10.89	12
16	control vs. dup 1000+IL-4+ds-RNA	1.000	1.300	-0.3005	0.08937	4	4	4.755	12
17	ds-RNA vs. IL-4+ds-RNA	1.287	1.688	-0.4013	0.08937	4	4	6.350	12
18	ds-RNA vs. dup 1000+IL-4+ds-RNA	1.287	1.300	-0.01341	0.08937	4	4	0.2122	12
19	IL-4+ds-RNA vs. dup 1000+IL-4+ds-RNA	1.688	1.300	0.3879	0.08937	4	4	6.138	12

Figure 5 DUPILUMAB+IL-13 PCR TSLP

NS	ds-RNA	IL-13+ds-RNA	dup 10+IL-13+ds-RNA	dup 100+IL-13+ds-RNA	dup 1000+IL-13+ds-RNA
1	24936,9949	51561,2636	29614,1694	22946,7148	19043,3543
1	24713,2992	82322,5471	33064,6039	19810,8066	26615,8865
1	3934,59656	13261,9013	8045,68756	5514,44508	5930,75475
1	3795,3047	14930,7602	14096,0175	4617,83209	5959,60088

Descriptive statistics	A<br>NS	B<br>ds-RNA	C<br>IL-13+ds-RNA	D<br>dup 10+IL-13+ds-RNA	E<br>dup 100+IL-13+ds-RNA	F<br>dup 1000+IL-13+ds-RNA
	Y	Y	Y	Y	Y	Y
Number of values	4	4	4	4	4	4
Minimum	1.000	3795	13262	8046	4618	5931
Maximum	1.000	24937	82323	33065	22947	26616
Range	0.000	21142	69061	25019	18329	20685
Mean	1.000	14345	40519	21205	13222	14387
Std. Deviation	0.000	12102	33001	12043	9512	10227
Std. Error of Mean	0.000	6051	16500	6021	4756	5113

	Normality and Lognormality Tests<br>Tabular results	A<br>NS<br>Y	B<br>ds-RNA<br>Y	C<br>IL-13+ds-RNA<br>Y	D<br>dup 10+IL-13+ds-RNA<br>Y	E<br>dup 100+IL-13+ds-RNA<br>Y	F<br>dup 1000+IL-13+ds-RNA<br>Y
1	**Test for normal distribution**						
2	**D'Agostino & Pearson test**						
3	K2	N too small	N too small	N too small	N too small	N too small	N too small
4	P value						
5	Passed normality test (alpha=0.05)?						
6	P value summary						
7							
8	**Anderson-Darling test**						
9	A2*	N too small	N too small	N too small	N too small	N too small	N too small
10	P value						
11	Passed normality test (alpha=0.05)?						
12	P value summary						
13							
14	**Shapiro-Wilk test**						
15	W	Invalid input d	0.7363	0.8779	0.8989	0.8262	0.8564
16	P value		0.0285	0.3297	0.4256	0.1581	0.2477
17	Passed normality test (alpha=0.05)?		No	Yes	Yes	Yes	Yes
18	P value summary		*	ns	ns	ns	ns
19							
20	**Kolmogorov-Smirnov test**						
21	KS distance	N too small	N too small	N too small	N too small	N too small	N too small
22	P value						
23	Passed normality test (alpha=0.05)?						
24	P value summary						
25							
26	**Number of values**	4	4	4	4	4	4

ANOVA results × Multiple comparisons ×

	Ordinary one-way ANOVA<br>ANOVA results					
1	Table Analyzed	5. Dupilumab and IL-13: PCR TSLP				
2	Data sets analyzed	A-F				
3	Distribution assumption	Normal (Gaussian)				
4						
5	**ANOVA summary**					
6	F	2.704				
7	P value	0.0542				
8	P value summary	ns				
9	Significant diff. among means (P < 0.05)?	No				
10	R squared	0.4289				
11						
12	**Brown-Forsythe test**					
13	F (DFn, DFd)	7.064 (5, 18)				
14	P value	0.0008				
15	P value summary	***				
16	Are SDs significantly different (P < 0.05)?	Yes				
17						
18	**Bartlett's test**					
19	Bartlett's statistic (corrected)					
20	P value					
21	P value summary					
22	Are SDs significantly different (P < 0.05)?					
23						
24	**ANOVA table**	**SS**	**DF**	**MS**	**F (DFn, DFd)**	**P value**
25	Treatment (between columns)	3549887223	5	709977445	F (5, 18) = 2.704	P=0.0542
26	Residual (within columns)	4726786604	18	262599256		
27	Total	8276673827	23			

ANOVA results | Multiple comparisons

Ordinary one-way ANOVA Multiple comparisons							
1	Number of families	1					
2	Number of comparisons per family	15					
3	Alpha	0.05					
4							
5	**Tukey's multiple comparisons test**	**Mean diff.**	**95.00% CI of diff.**	**Below threshold?**	**Summary**	**Adjusted P Value**	
6	NS vs. ds-RNA	-14344	-50760 to 22072	No	ns	0.8060	A-B
7	NS vs. IL-13+ds-RNA	-40518	-76934 to -4102	Yes	*	0.0243	A-C
8	NS vs. dup 10+IL-13+ds-RNA	-21204	-57620 to 15212	No	ns	0.4609	A-D
9	NS vs. dup 100+IL-13+ds-RNA	-13221	-49637 to 23194	No	ns	0.8523	A-E
10	NS vs. dup 1000+IL-13+ds-RNA	-14386	-50802 to 22029	No	ns	0.8042	A-F
11	ds-RNA vs. IL-13+ds-RNA	-26174	-62590 to 10242	No	ns	0.2503	B-C
12	ds-RNA vs. dup 10+IL-13+ds-RNA	-6860	-43276 to 29556	No	ns	0.9898	B-D
13	ds-RNA vs. dup 100+IL-13+ds-RNA	1123	-35293 to 37538	No	ns	>0.9999	B-E
14	ds-RNA vs. dup 1000+IL-13+ds-RNA	-42.35	-36458 to 36373	No	ns	>0.9999	B-F
15	IL-13+ds-RNA vs. dup 10+IL-13+ds-RNA	19314	-17102 to 55730	No	ns	0.5574	C-D
16	IL-13+ds-RNA vs. dup 100+IL-13+ds-RNA	27297	-9119 to 63713	No	ns	0.2140	C-E
17	IL-13+ds-RNA vs. dup 1000+IL-13+ds-RNA	26132	-10284 to 62548	No	ns	0.2517	C-F
18	dup 10+IL-13+ds-RNA vs. dup 100+IL-13+ds-RNA	7983	-28433 to 44399	No	ns	0.9800	D-E
19	dup 10+IL-13+ds-RNA vs. dup 1000+IL-13+ds-RNA	6818	-29598 to 43234	No	ns	0.9901	D-F
20	dup 100+IL-13+ds-RNA vs. dup 1000+IL-13+ds-RNA	-1165	-37581 to 35251	No	ns	>0.9999	E-F

Figure 5 DUPILUMAB+IL-13 PCR IL-8

NS	ds-RNA	IL-13+ds-RNA	dup 10+IL-13+ds-RNA	dup 100+IL-13+ds-RNA	dup 1000+IL-13+ds-RNA
1	74,6463597	108,3834	110,968008	106,965371	65,0284892
1	70,5219274	104,329259	68,5459727	57,8802802	48,4357374
1	32,9224547	167,614134	94,3532299	48,1011673	38,0810143

Descriptive statistics		A	B	C	D	E	F
		NS	ds-RNA	IL-13+ds-RNA	dup 10+IL-13+ds-RNA	dup 100+IL-13+ds-RNA	dup 1000+IL-13+ds-RNA
		Y	Y	Y	Y	Y	Y
1	Number of values	3	3	3	3	3	3
2							
3	Minimum	1.000	32.92	104.3	68.55	48.10	38.08
4	Maximum	1.000	74.65	167.6	111.0	107.0	65.03
5	Range	0.000	41.72	63.28	42.42	58.86	26.95
6							
7	Mean	1.000	59.36	126.8	91.29	70.98	50.52
8	Std. Deviation	0.000	22.99	35.43	21.38	31.54	13.59
9	Std. Error of Mean	0.000	13.27	20.45	12.34	18.21	7.848

Normality and Lognormality Tests<br>Tabular results	A<br>NS	B<br>ds-RNA	C<br>IL-13+ds-RNA	D<br>dup 10+IL-13+ds-RNA	E<br>dup 100+IL-13+ds-RNA	F<br>dup 1000+IL-13+ds-RNA
	Y	Y	Y	Y	Y	Y
**Test for normal distribution**						
**D'Agostino & Pearson test**						
K2	N too small	N too small	N too small	N too small	N too small	N too small
P value						
Passed normality test (alpha=0.05)?						
P value summary						
**Anderson-Darling test**						
A2*	N too small	N too small	N too small	N too small	N too small	N too small
P value						
Passed normality test (alpha=0.05)?						
P value summary						
**Shapiro-Wilk test**						
W	Invalid input d	0.8233	0.7978	0.9846	0.8706	0.9825
P value		0.1715	0.1093	0.7623	0.2972	0.7463
Passed normality test (alpha=0.05)?		Yes	Yes	Yes	Yes	Yes
P value summary		ns	ns	ns	ns	ns
**Kolmogorov-Smirnov test**						
KS distance	N too small	N too small	N too small	N too small	N too small	N too small
P value						
Passed normality test (alpha=0.05)?						
P value summary						
**Number of values**	3	3	3	3	3	3

ANOVA results × Multiple comparisons ×

Ordinary one-way ANOVA<br>ANOVA results					
Table Analyzed	5. Dupilumab and IL-13: PCR IL-8				
Data sets analyzed	A-F				
Distribution assumption	Normal (Gaussian)				
**ANOVA summary**					
F	9.330				
P value	0.0008				
P value summary	***				
Significant diff. among means (P < 0.05)?	Yes				
R squared	0.7954				
**Brown-Forsythe test**					
F (DFn, DFd)	0.4383 (5, 12)				
P value	0.8136				
P value summary	ns				
Are SDs significantly different (P < 0.05)?	No				
**Bartlett's test**					
Bartlett's statistic (corrected)					
P value					
P value summary					
Are SDs significantly different (P < 0.05)?					
**ANOVA table**	**SS**	**DF**	**MS**	**F (DFn, DFd)**	**P value**
Treatment (between columns)	26593	5	5319	F (5, 12) = 9.330	P=0.0008
Residual (within columns)	6841	12	570.0		
Total	33433	17			

ANVOA results | Multiple comparisons

Ordinary one-way ANOVA Multiple comparisons							
1	Number of families	1					
2	Number of comparisons per family	15					
3	Alpha	0.05					
4							
5	**Tukey's multiple comparisons test**	**Mean diff.**	**95.00% CI of diff.**	**Below threshold?**	**Summary**	**Adjusted P Value**	
6	NS vs. ds-RNA	-58.36	-123.8 to 7.117	No	ns	0.0915	A-B
7	NS vs. IL-13+ds-RNA	-125.8	-191.3 to -60.30	Yes	***	0.0004	A-C
8	NS vs. dup 10+IL-13+ds-RNA	-90.29	-155.8 to -24.81	Yes	**	0.0059	A-D
9	NS vs. dup 100+IL-13+ds-RNA	-69.98	-135.5 to -4.502	Yes	*	0.0339	A-E
10	NS vs. dup 1000+IL-13+ds-RNA	-49.52	-115.0 to 15.97	No	ns	0.1869	A-F
11	ds-RNA vs. IL-13+ds-RNA	-67.41	-132.9 to -1.932	Yes	*	0.0423	B-C
12	ds-RNA vs. dup 10+IL-13+ds-RNA	-31.93	-97.41 to 33.55	No	ns	0.5918	B-D
13	ds-RNA vs. dup 100+IL-13+ds-RNA	-11.62	-77.10 to 53.86	No	ns	0.9893	B-E
14	ds-RNA vs. dup 1000+IL-13+ds-RNA	8.849	-56.63 to 74.33	No	ns	0.9969	B-F
15	IL-13+ds-RNA vs. dup 10+IL-13+ds-RNA	35.49	-29.99 to 101.0	No	ns	0.4889	C-D
16	IL-13+ds-RNA vs. dup 100+IL-13+ds-RNA	55.79	-9.687 to 121.3	No	ns	0.1132	C-E
17	IL-13+ds-RNA vs. dup 1000+IL-13+ds-RNA	76.26	10.78 to 141.7	Yes	*	0.0197	C-F
18	dup 10+IL-13+ds-RNA vs. dup 100+IL-13+ds-RNA	20.31	-45.17 to 85.79	No	ns	0.8947	D-E
19	dup 10+IL-13+ds-RNA vs. dup 1000+IL-13+ds-RNA	40.77	-24.71 to 106.3	No	ns	0.3521	D-F
20	dup 100+IL-13+ds-RNA vs. dup 1000+IL-13+ds-RNA	20.47	-45.01 to 85.95	No	ns	0.8917	E-F
21							

Figure 5 DUPILUMAB+IL-13 WB TSLP

control	ds-RNA	IL-13+ds-RNA	dup 1000+IL-13+ds-RNA
1	1,257150645	1,610115813	1,314489675
1	1,395757	1,814648933	1,186019519
1	1,266677938	1,611003805	1,354202965

Descriptive statistics		A	B	C	D
		control	ds-RNA	IL-13+ds-RNA	dup 1000+IL-13+ds-RNA
		Y	Y	Y	Y
1	Number of values	3	3	3	3
2					
3	Minimum	1.000	1.257	1.610	1.186
4	Maximum	1.000	1.396	1.815	1.354
5	Range	0.000	0.1386	0.2045	0.1682
6					
7	Mean	1.000	1.307	1.679	1.285
8	Std. Deviation	0.000	0.07742	0.1178	0.08791
9	Std. Error of Mean	0.000	0.04470	0.06803	0.05075

Normality and Lognormality Tests<br>Tabular results	A	B	C	D
	control	ds-RNA	IL-13+ds-RNA	dup 1000+IL-13+ds-RNA
	Y	Y	Y	Y
**Test for normal distribution**				
**D'Agostino & Pearson test**				
K2	N too small	N too small	N too small	N too small
P value				
Passed normality test (alpha=0.05)?				
P value summary				
**Anderson-Darling test**				
A2*	N too small	N too small	N too small	N too small
P value				
Passed normality test (alpha=0.05)?				
P value summary				
**Shapiro-Wilk test**				
W	Invalid input d	0.8013	0.7533	0.9151
P value		0.1176	0.0072	0.4352
Passed normality test (alpha=0.05)?		Yes	No	Yes
P value summary		ns	**	ns
**Kolmogorov-Smirnov test**				
KS distance	N too small	N too small	N too small	N too small
P value				
Passed normality test (alpha=0.05)?				
P value summary				
**Number of values**	3	3	3	3

ANOVA results × | Multiple comparisons ×

Ordinary one-way ANOVA<br>ANOVA results					
Table Analyzed	5. Dupilumab and IL13: Protein TSLP				
Data sets analyzed	A-D				
**ANOVA summary**					
F	33.67				
P value	<0.0001				
P value summary	****				
Significant diff. among means (P < 0.05)?	Yes				
R squared	0.9266				
**Brown-Forsythe test**					
F (DFn, DFd)	0.4579 (3, 8)				
P value	0.7192				
P value summary	ns				
Are SDs significantly different (P < 0.05)?	No				
**Bartlett's test**					
Bartlett's statistic (corrected)					
P value					
P value summary					
Are SDs significantly different (P < 0.05)?					
**ANOVA table**	**SS**	**DF**	**MS**	**F (DFn, DFd)**	**P value**
Treatment (between columns)	0.6971	3	0.2324	F (3, 8) = 33.67	P<0.0001
Residual (within columns)	0.05521	8	0.006902		
Total	0.7523	11			
**Data summary**					
Number of treatments (columns)	4				
Number of values (total)	12				

ANOVA results | **Multiple comparisons**

	Ordinary one-way ANOVA<br>Multiple comparisons								
1	Number of families	1							
2	Number of comparisons per family	6							
3	Alpha	0.05							
4									
5	**Tukey's multiple comparisons test**	**Mean Diff.**	**95.00% CI of diff.**	**Below threshold?**	**Summary**	**Adjusted P Value**			
6	control vs. ds-RNA	-0.3065	-0.5237 to -0.08931	Yes	**	0.0084	A-B		
7	control vs. IL-13+ds-RNA	-0.6786	-0.8958 to -0.4614	Yes	****	<0.0001	A-C		
8	control vs. dup 1000+IL-13+ds-RNA	-0.2849	-0.5021 to -0.06769	Yes	*	0.0128	A-D		
9	ds-RNA vs. IL-13+ds-RNA	-0.3721	-0.5893 to -0.1548	Yes	**	0.0026	B-C		
10	ds-RNA vs. dup 1000+IL-13+ds-RNA	0.02162	-0.1956 to 0.2388	No	ns	0.9880	B-D		
11	IL-13+ds-RNA vs. dup 1000+IL-13+ds-RNA	0.3937	0.1765 to 0.6109	Yes	**	0.0018	C-D		
12									
13	**Test details**	**Mean 1**	**Mean 2**	**Mean Diff.**	**SE of diff.**	**n1**	**n2**	**q**	**D**
14	control vs. ds-RNA	1.000	1.307	-0.3065	0.06783	3	3	6.391	8
15	control vs. IL-13+ds-RNA	1.000	1.679	-0.6786	0.06783	3	3	14.15	8
16	control vs. dup 1000+IL-13+ds-RNA	1.000	1.285	-0.2849	0.06783	3	3	5.940	8
17	ds-RNA vs. IL-13+ds-RNA	1.307	1.679	-0.3721	0.06783	3	3	7.757	8
18	ds-RNA vs. dup 1000+IL-13+ds-RNA	1.307	1.285	0.02162	0.06783	3	3	0.4509	8
19	IL-13+ds-RNA vs. dup 1000+IL-13+ds-RNA	1.679	1.285	0.3937	0.06783	3	3	8.208	8
20									

Figure 5 DUPILUMAB+IL-13 WB IL-8

control	ds-RNA	IL-13+ds-RNA	dup 1000+IL-13+ds-RNA
1	1,36962184	1,66915404	1,456545734
1	1,33328773	1,62517313	1,486237554
1	1,26463883	1,62890448	1,240965056

	Descriptive statistics	A<br>control<br>Y	B<br>ds-RNA<br>Y	C<br>IL-13+ds-RNA<br>Y	D<br>dup 1000+IL-13+ds-RNA<br>Y
1	Number of values	3	3	3	3
2					
3	Minimum	1.000	1.265	1.625	1.241
4	Maximum	1.000	1.370	1.669	1.486
5	Range	0.000	0.1050	0.04398	0.2453
6					
7	Mean	1.000	1.323	1.641	1.395
8	Std. Deviation	0.000	0.05331	0.02439	0.1339
9	Std. Error of Mean	0.000	0.03078	0.01408	0.07729

Normality and Lognormality Tests
Tabular results

		A	B	C	D
		control	ds-RNA	IL-13+ds-RNA	dup 1000+IL-13+ds-RNA
		Y	Y	Y	Y
1	**Test for normal distribution**				
2	**D'Agostino & Pearson test**				
3	K2	N too small	N too small	N too small	N too small
4	P value				
5	Passed normality test (alpha=0.05)?				
6	P value summary				
7					
8	**Anderson-Darling test**				
9	A2*	N too small	N too small	N too small	N too small
10	P value				
11	Passed normality test (alpha=0.05)?				
12	P value summary				
13					
14	**Shapiro-Wilk test**				
15	W	Invalid input d	0.9694	0.8131	0.8393
16	P value		0.6641	0.1463	0.2122
17	Passed normality test (alpha=0.05)?		Yes	Yes	Yes
18	P value summary		ns	ns	ns
19					
20	**Kolmogorov-Smirnov test**				
21	KS distance	N too small	N too small	N too small	N too small
22	P value				
23	Passed normality test (alpha=0.05)?				
24	P value summary				
25					
26	**Number of values**	3	3	3	3

ANOVA results × | Multiple comparisons ×

Ordinary one-way ANOVA
ANOVA results

1	Table Analyzed	5. Dupilumab and IL13: Protein IL-8				
2	Data sets analyzed	A-D				
3	Distribution assumption	Normal (Gaussian)				
4						
5	**ANOVA summary**					
6	F	39.24				
7	P value	<0.0001				
8	P value summary	****				
9	Significant diff. among means (P < 0.05)?	Yes				
10	R squared	0.9364				
11						
12	**Brown-Forsythe test**					
13	F (DFn, DFd)	0.9935 (3, 8)				
14	P value	0.4437				
15	P value summary	ns				
16	Are SDs significantly different (P < 0.05)?	No				
17						
18	**Bartlett's test**					
19	Bartlett's statistic (corrected)					
20	P value					
21	P value summary					
22	Are SDs significantly different (P < 0.05)?					
23						
24	**ANOVA table**	**SS**	**DF**	**MS**	**F (DFn, DFd)**	**P value**
25	Treatment (between columns)	0.6286	3	0.2095	F (3, 8) = 39.24	P<0.0001
26	Residual (within columns)	0.04271	8	0.005339		
27	Total	0.6713	11			

ANOVA results | Multiple comparisons

Ordinary one-way ANOVA
Multiple comparisons

1	Number of families	1							
2	Number of comparisons per family	6							
3	Alpha	0.05							
4									
5	**Tukey's multiple comparisons test**	**Mean diff.**	**95.00% CI of diff.**	**Below threshold?**	**Summary**	**Adjusted P Value**			
6	control vs. ds-RNA	-0.3225	-0.5136 to -0.1315	Yes	**	0.0028	A-B		
7	control vs. IL-13+ds-RNA	-0.6411	-0.8321 to -0.4500	Yes	****	<0.0001	A-C		
8	control vs. dup 1000+IL-13+ds-RNA	-0.3946	-0.5856 to -0.2035	Yes	***	0.0008	A-D		
9	ds-RNA vs. IL-13+ds-RNA	-0.3186	-0.5096 to -0.1275	Yes	**	0.0031	B-C		
10	ds-RNA vs. dup 1000+IL-13+ds-RNA	-0.07207	-0.2631 to 0.1190	No	ns	0.6392	B-D		
11	IL-13+ds-RNA vs. dup 1000+IL-13+ds-RNA	0.2465	0.05544 to 0.4375	Yes	*	0.0140	C-D		
12									
13	**Test details**	**Mean 1**	**Mean 2**	**Mean diff.**	**SE of diff.**	**n1**	**n2**	**q**	**DF**
14	control vs. ds-RNA	1.000	1.323	-0.3225	0.05966	3	3	7.645	8
15	control vs. IL-13+ds-RNA	1.000	1.641	-0.6411	0.05966	3	3	15.20	8
16	control vs. dup 1000+IL-13+ds-RNA	1.000	1.395	-0.3946	0.05966	3	3	9.353	8
17	ds-RNA vs. IL-13+ds-RNA	1.323	1.641	-0.3186	0.05966	3	3	7.551	8
18	ds-RNA vs. dup 1000+IL-13+ds-RNA	1.323	1.395	-0.07207	0.05966	3	3	1.708	8
19	IL-13+ds-RNA vs. dup 1000+IL-13+ds-RNA	1.641	1.395	0.2465	0.05966	3	3	5.843	8
